# Ambient temperature and the variability between neighbouring days impacts in-patient hospitalizations in the United Kingdom

**DOI:** 10.1038/s43856-025-01355-y

**Published:** 2026-01-26

**Authors:** Ka Yan Lai, Chris Webster, John Gallacher, Chinmoy Sarkar

**Affiliations:** 1 Healthy High Density Cities Lab, HKUrbanLab, The University of Hong Kong, Hong Kong Special Administrative Region, Hong Kong, China; 2 Institute for Climate and Carbon Neutrality, The University of Hong Kong, Hong Kong Special Administrative Region, Hong Kong, China; 3 Department of Urban Planning & Design, Faculty of Architecture, The University of Hong Kong, Hong Kong Special Administrative Region, Hong Kong, China; 4 Urban Systems Institute, The University of Hong Kong, Hong Kong Special Administrative Region, Hong Kong, China; 5 Department of Psychiatry, University of Oxford, Oxford, UK

**Keywords:** Public health, Disease prevention

## Abstract

**Background:**

Acute health effects of temperature extremes and variability in temperate zones has been rarely quantified. We examine the associations of ambient temperature and temperature change between neighbouring days with all-cause and cause-specific hospitalizations.

**Methods:**

Daily hospital admission data were identified through hospital record linkage with UK Biobank, a cohort of half-a-million participants during 2006-2022. Temperature exposure was measured at 1×1 Km^2^ spatial resolution based on participants’ residential addresses. We used a time-stratified case-crossover design to examine short-term associations of ambient temperature and change in temperature between neighbouring days with all-cause and cause-specific hospitalizations.

**Results:**

We identify 709,052 warm-season hospitalizations and 676,686 cold-season hospitalizations. During warm season, high temperature cumulated over lag 0-3 days is associated with 9% [odds ratio (OR) = 1.09, 95% confidence interval (CI) = 1.02, 1.16] and 18% (OR = 1.18, 95% CI = 1.05, 1.34) higher odds of hospitalizations for renal disease and heat-related illness, respectively. During cold season, high temperature is associated with 4% (OR = 1.04, 95% CI = 1.01, 1.06) higher odds of overall hospitalizations from any cause, and also for cardiovascular disease (OR = 1.06, 95% CI = 1.02, 1.09), respiratory disease (OR = 1.05, 95% CI = 1.00, 1.11), mental disorders (OR = 1.08, 95% CI = 1.00, 1.16) and heat-related illness (OR = 1.25, 95% CI = 1.05, 1.48). We observe more pronounced associations between ambient temperature and overall hospitalization among subgroups residing in the most deprived neighbourhoods and with the least greenspace coverage during both warm and cold seasons.

**Conclusions:**

Our findings suggest the need for multilevel mitigation and adaptation strategies for strengthening individual and urban resilience to minimize adverse health effects attributable to temperature extremes.

## Plain Language Summary

Temperatures are increasing globally. Information about the health impact of temperature extremes and its day-to-day variations can help inform climate mitigation and adaptation policies to improve health. We used 1.38 million hospitalization records from the UK and found there was an association between ambient temperature and all-cause and cause-specific hospitalizations, which varied by season. We also found more pronounced associations between ambient temperature and overall hospitalization among people living in the most deprived neighbourhoods and those with the least greenspace coverage during both warm and cold seasons. These findings could be used to inform targeted mitigation and adaptation strategies to improve health during temperature extremes.

## Abstract (web summary)

Lai et al. use hospitalization records from the UK to examine associations between ambient temperature and changing temperature with all-cause and cause-specific hospitalizations. There is seasonal variation in associations with more pronounced effects in people living in deprived neighbourhoods and with low greenspace coverage.

## Introduction

Since the beginning of global climatic archive in 1850, the warmest decade has only been recently recorded over 2015–24^1^. Anthropogenic greenhouse gas emissions associated with increasing demand for material and energy resources have been the primary driver of global warming phenomena^2^. In general, this has resulted in warmer mean climatic patterns in the northern hemisphere^3^, characterized by marked seasonal shifts in the form of early onset and lengthened summers and shortened and milder winters^4^. There has also been growing evidence of increase in the frequency of extreme temperature events^5^. Diurnal variability in mean temperature has shown a downward trend^6^, while it has increased over longer time scales^7^. These climatic changes have been associated with significant economic and societal impacts^8,9^, including population health.

Climate and human health are intrinsically interlinked. Accumulating scientific evidence over the past two decades have consistently established links between climate change and adverse human health^10,11^. The urgent necessity for actionable policies and interventions to ameliorate climate-induced health effects is reflected across various governmental health policy agendas globally^12,13^. Short-term exposure to extreme ambient temperature has been known to directly cause adverse health outcome and hospitalization as well as indirectly act as a trigger for them resulting in subsequent hospitalization. Previous research have reported on adverse associations of extreme ambient temperature with mortality^14,15^, physical health including cardiovascular^16^, respiratory^17^ and renal diseases^18^, and mental health disorders^19^. The duration of short-term exposure in previous studies varied between examining the effects of temperature extremes measured over longer lag periods (such as 14 days) for quantifying accumulated health effects^20,21^ to relatively shorter periods (such as 3 days) for acute health conditions including heart failure^22^ and preclinical alterations in heart failure status^23^. Growing attention has also been drawn towards identifying population vulnerability to acute effects for tailor-made multi-level interventions^24^. Effect heterogeneity by individual-level characteristics such as age, sex and physical fitness can provide the much needed evidence for identifying individuals and sub-groups who are likely to be vulnerable to climatic extremes, so that they can be informed of preventive measures to mitigate climate-induced adverse health effects^24–26^. Neighbourhood-level heterogeneity such as neighbourhood deprivation^27,28^ and greenness^29^ are of value to inform social and urban policies for strengthening community support and improving population health.

Evidence has also emerged of anthropogenic climate change induced anomalies in day-to-day temperatures and its short-term acute effects on health. From a healthcare policy perspective, this remains an active area of investigation, given the importance of objectively measuring climate-induced burdens on healthcare systems and planning health adaptations. A few studies have employed mortality records and emergency department datasets and used case-crossover design to examine associations between ambient temperature at specific lag days of hospitalization and healthcare burdens measured in terms of daily-aggregated counts of mortality^20^ and morbidity^30–32^. Individual-level evidence of short-term associations with hospitalization outcomes remain very scarce^33,34^. Furthermore, some of these studies are single-city^35^ and several have assessed temperature at aggregated levels of city^20^, census geographies^30,31^ or the nearest meteorological monitoring station^34,35^ resulting in limited spatial exposure variability. Outcome-wide evidence linking temperature change between neighbouring days and morbidity, which provide a nuanced understanding of links between day-to-day temperature fluctuations and health has been lacking. In this study, we employed data from a UK-wide cohort with linkage to in-patient health records to examine associations of ambient temperature and change in temperature between neighbouring days with all-cause and cause-specific hospitalizations using individual-level case-crossover design. We also investigated effects by age group, sex, frailty, physical activity, neighbourhood deprivation, residential greenspace and climatic zones of UK. In brief, this study shows seasonal variation in the association between ambient temperature and all-cause and cause-specific hospitalizations. The study reports that higher temperature is associated with higher odds of hospitalizations for renal disease and heat-related illness during warm season; and higher odds of overall hospitalizations from any cause, CVD, respiratory disease, mental disorders and heat-related illness during cold season. It also identifies more pronounced associations between ambient temperature and overall hospitalization among people living in the most deprived neighbourhoods and those with the least greenspace coverage during both warm and cold seasons. These findings are of value to inform mitigation and adaptation strategies to minimize health risks attributable to climate extremes.

## Methods

### Study population

This individual-level time-stratified case-crossover study was nested within the UK Biobank, an UK-wide cohort of 0.5 million adults aged 37–73 years at baseline^36^. UK Biobank recruited half-a-million participants who were registered with the National Health Service over March 13, 2006 up to October 1, 2010, with a response rate of 5.5% at baseline. Assessments were conducted in 22 assessment centres located in cities across United Kingdom and participants resided within a 25-mile radius of these assessment centres. Participants answered online questionnaire surveys and undertook anthropogenic examinations, with detailed measurement protocols available in Data Showcase (https://biobank.ndph.ox.ac.uk/showcase/). Record linkage was established with hospital episode data and death register. The detailed cohort protocol is available elsewhere^37^. All participants provided informed consent prior to the assessments. This research was conducted with the UK Biobank’s permission of data access upon our request under an approved application number of 11730. UK Biobank obtained ethical approval from the NHS National Research Ethics Service (11/NW/0382) and renewed every 5 years (16/NW/0274 and 21/NW/0157). The study also received ethical approval from The University of Hong Kong’s Human Research Ethics Committee (ref: EA220302).

This study is restricted to adult UK Biobank participants who underwent inpatient hospitalization after entry in to the cohort from March 13, 2006 onwards until the end of follow-up on December 31, 2022. Dates and causes of hospitalization for each participant was obtained through record linkage with Hospital Episode Statistics Admitted Patient Care (England), Patient Episode Database (Wales), and General/ Acute Inpatient and Day Case-Scottish Morbidity Record (Scotland). We identified a range of causes of inpatient hospitalizations from the hospital episode data using ICD 10 (International Classification of Diseases –10th revision) diagnostic codes (Supplementary Table 1). These included hospitalizations owing to any cause (ICD-10: A00-Y98), cardiovascular disease (ICD-10: I00-I99), chronic respiratory disease (ICD-10: J30-J98, excluding J69, J96), renal disease (ICD-10: N00-N21, N25-N27)), mental health disorders (ICD-10: F01-F99) and heat related disorders (E86-E87, T67, X30).

### Assessment of climatic exposures

We used the UK Met Office’s HadUK-Grid dataset to calculate the daily mean ambient temperature (°C) and rainfall (mm) available at: https://www.metoffice.gov.uk/research/climate/maps-and-data/data/haduk-grid/datasets. HadUK-Grid is the nation-wide archive of climatic variables interpolated from the in-situ land surface measurements and meteorological station observations. Several previous studies have assigned climatic observations such as temperature derived from the meteorological station nearest to participant’s census geography of residence resulting in limited spatial resolution to reliably detect health effects. Exposure measurements based on few unevenly distributed monitoring stations has been known to induce measurement error, underestimating the effect sizes^38,39^. To account for spatial variability in data available from monitoring stations and geographic variability in environmental factors that influence temperature, exposure data were generated following a two-stage procedure^40,41^. Firstly, regression models of association of normalized temperature values with geographic variables (including latitude, longitude, terrain elevation and shape, proportion of open water including lake and sea, and urban land use) were developed. In the second step, interpolation was conducted on regression residuals onto the standardized 1 × 1 Km^2^ Ordnance Survey’s National grids using the inverse-distance weighted averaging (IDW) method. The daily root mean square error (RMSE) for the observed station dataset and generated gridded data for maximum and minimum temperature was 1.06 and 1.27 °C respectively. Mean temperature was calculated by averaging the daily minimum and maximum temperatures. Gridded daily rainfall data was generated through interpolation upon the normalized rainfall values itself. To define individual-level exposure in the present study, we first extracted daily climatic exposure data over the study period of 2006–22 and spatially linked them to UK Biobank participants’ geocoded home addresses. The climatic exposure data at such refined spatial resolution have also been employed to study heat-induced health impact previously^42^. For each hospitalization record located in grid *j*, individual-level temperature exposure was defined as the daily mean ambient temperature based on each participant’s residential address for each day of the warm and cold seasons. The analysis was conducted by season throughout. Warm season was defined as periods between June and September, whereas cold season was defined as periods between November and February. Temperature change between neighbouring days (TCN) was calculated as the difference between the mean temperatures on a day minus that of the previous day, with positive values indicating temperature increment and negative values decrement.

### Other variables

Other study variables included age, sex, leisure time physical activity and frailty. Area-level variables included Townsend index as a measure of neighbourhood deprivation, residential density^43^, total residential greenspace and natural greenspace measured within 1-Km street catchment. Frailty^44^ was defined as a composite index constructed from five traits including weight loss, exhaustion, low grip strength, low physical activity, and slow gait speed (see Supplementary Method for more details). Participants who did not meet any of the criteria were classified as non-frail, whereas meeting any one or two were classified as pre-frail. Fulfillment of three or more criteria were classified as frail. Residential greenspace was calculated by the sum of natural greenspace, urban parks, allotments and cemeteries.

### Statistical analysis

We conducted a time-stratified case-crossover study^45^ nested within the UK Biobank cohort to estimate short-term association of daily mean ambient temperature and TCN with any cause and cause-specific in-patient hospitalization. In this design, each cohort participant undergoing hospitalization over the study period acted as their own control and the relative risk of hospitalization are estimated by comparing the effect of daily ambient temperature and TCN measured on the case day relative to the control days. The ‘case day’ was defined as the date of hospitalization and the temperature exposure was measured on this day and at specific lag periods for each participant. A time-stratified approach was applied to select ‘control days’ matched by the calendar year, month and day of the week at a matching ratio of 3 or 4 controls per case. For example, if a patient is hospitalized on Sep 12, 2021 (case day), a total of 3 control days (Sep 5, Sep 19 and Sep 26, 2021) are selected. This helped adjust confounding by seasonal and long-term trends and by day of the week. The strength of this design also originates from its inherent ability to eliminate control-selection bias by controlling for both known and unknown time-invariant confounders (such as age, sex, socio-demographics) and to a great extent overcoming the issue of residual confounding^30,46^.

We used conditional logistic regression models to quantify the associations of daily ambient temperature and TCN with the odds of any-cause and cause-specific hospitalizations. The distributed lag nonlinear modelling (DLNM) framework was incorporated into the conditional logistic regression models^14^ to examine non-linear and lagged associations. Both non-linear exposure-response function and non-linear lag response function for ambient temperature and TCN were used. In this study, we modelled the exposure-response function using a quadratic B spline with a single knot placed at the 50^th^ centile of the mean temperature or TCN distribution, informed by a prior nation-wide case-crossover study assessing the impacts of heat on emergency department visits for multiple causes^30^. The lag response function was modelled using a natural cubic B spline with two knots placed at equal intervals by the log of lags to allow for flexible lag effects for each lag day with a maximum of up to three days prior to the hospitalization. The choice of three lag days was informed by prior evidence showing that the risks were limited to the first few days (i.e., 3 days^22^, or 5 days^30^). Additionally, our initial data exploration allowing for lag effects with a maximum of up to 7 days found that the temperature effects were more pronounced on the first two lag days during warm season and the day of diagnosis for cold season (Supplementary Fig. 1). The main models for mean temperature and TCN are presented below:

1a $$E\left({Y}_{i}\right)={cb}.({{Temp}}_{{ij}}^{d},{lag}=3)+{ns}.({{Rainfall}}_{{ij}}^{d},{df}=3)+{{stratum}}_{i}+{{holiday}}_{{ij}}^{d}\ldots$$

1b $$E\left({Y}_{i}\right)={cb}.({{TCN}}_{{ij}}^{d},{lag}=3)+{ns}.({{Rainfall}}_{{ij}}^{d},{df}=3)+{{stratum}}_{i}+{{holiday}}_{{ij}}^{d}\ldots$$

where $E\left({Y}_{i}\right)$ represents a binary variable for a participant presenting as a case *i* of hospitalization or its controls; ${cb}.{{Temp}}_{{ij}}^{d}$ and ${cb}.{{TCN}}_{{ij}}^{d}$ are the cross-basis functions for daily mean temperature and TCN for each $i$ over three lag days from the day of hospitalization $d$ (lag 0-3 days) in grid $j$; ${ns}.{{Rainfall}}_{{ij}}^{d}$ is the natural cubic spline with 3 degrees of freedom (${df}$) for mean daily rainfall exposure for each $i$ measured over lag 0-3 days in grid $j$; ${{stratum}}_{i}$ refers to the paired clusters matched by the same year, month, and day of the week for each $i$, and ${{holiday}}_{{ij}}^{d}$ is a binary variable to account for holiday effects. Rainfall was inserted in to the model to account for its confounding effect. Rainfall can influence human behaviours (such as lowering physical activities) and indoor exposures with poorer environmental quality (such as molds and fungi). Exposure-response curves were plotted based on a truncated dataset bounded within the 1^st^ and 99^th^ percentile of temperature or TCN distribution to minimize noise stemming from the small sample size at the two extremities of the exposure spectrum. To enhance interpretation of the results, we identified *high temperature* as days with a mean temperature at the 95^th^ percentile of the exposure distribution (as proxy of extreme exposure) and reported odds ratio (OR) and 95% confidence intervals (CI) for days of high temperature relative to the referent temperature. The referent temperature was equivalent to the temperature percentile at which minimum morbidity risk occurred, as identified from the exposure-response curve. For mean ambient temperature, the referent temperature corresponded to the first percentile of distribution. For TCN, the referent temperature was set at 0 °C, equivalent to no temperature change between neighbouring days, and we reported the ORs and CIs associated with the *positive TCN* (95^th^ percentile). Primary analysis examined independent associations of temperature and TCN on hospitalizations for any cause by warm and cold seasons to capture potential seasonal variations. Secondary analysis examined cause-specific hospitalizations including cardiovascular disease, respiratory disease, renal disease, mental health disorders and heat related illness. We further examined the effects of *moderately high temperature* and *moderately positive TCN* on hospitalization by setting of daily mean temperature and TCN at the 90^th^ percentile of the exposure distribution.

Several sensitivity tests were conducted to examine robustness of our results. Firstly, we reran the main analysis by changing key model parameters in the cross-basis function, including modelling exposure-response functions using a quadratic B spline with one knot placed at 75^th^ percentile^47^, two knots placed at 50^th^ and 90^th^ percentiles^48^ and three knots placed at 10^th^, 50^th^ and 90^th^ percentiles^49^, and modelling the lag response function using a natural cubic spline with one knot placed at equal intervals by the log of lags with a maximum of three days. Secondly, to examine if the results were robust to the change of parameters in covariates, we repeated the main analysis by fitting rainfall measured over 0–3 lag days by use of a natural cubic spline with 4 *df*. Thirdly, to strengthen confidence in the main analysis, we performed an additional analysis by using a longer lag period of 10 days. Fourthly, since the study period (2006–2022) included the duration of COVID-19 pandemic, we conducted subgroup analysis, dividing by the time of diagnosis into pre-COVID (2006–2019) and during-COVID (2020–2022) periods to examine potential differences in associations before and during the pandemic. Fifthly, to examine potential effects among participants undergoing multiple hospitalizations over the study period, we reran the main analysis by including only the first hospitalization record for each individual and excluding subsequent hospitalizations. Sixthly, given the temperature variation across geographical regions, we reran the main analysis using temperature percentile thresholds derived from specific climatic regions of UK. We examined potential effect modifications by population subgroups such as age (≥65 years vs < 65 years), sex (women *vs* men), frailty (frail *vs* non-frail), physical activity (normal or high *vs* low), and environmental exposures including neighbourhood deprivation (tertiles), residential greenspace (tertiles), natural greenspace (tertiles), and climatic regions of UK (South East and West, East and West Midlands, North East and West, Yorkshire and the Humber, Wales, and Scotland). Wald test was employed to examine if the associations were homogeneous across subgroups^50^.

All statistical analysis were conducted using R version 4.4.2. Conditional logistic regression was performed using the “survival” package version 3.8.3 and the distributed lag non-linear model was performed using the “dlnm” package version 2.4.7.

### Reporting summary

Further information on research design is available in the Nature Portfolio Reporting Summary linked to this article.

## Results

**Fig. 1:**
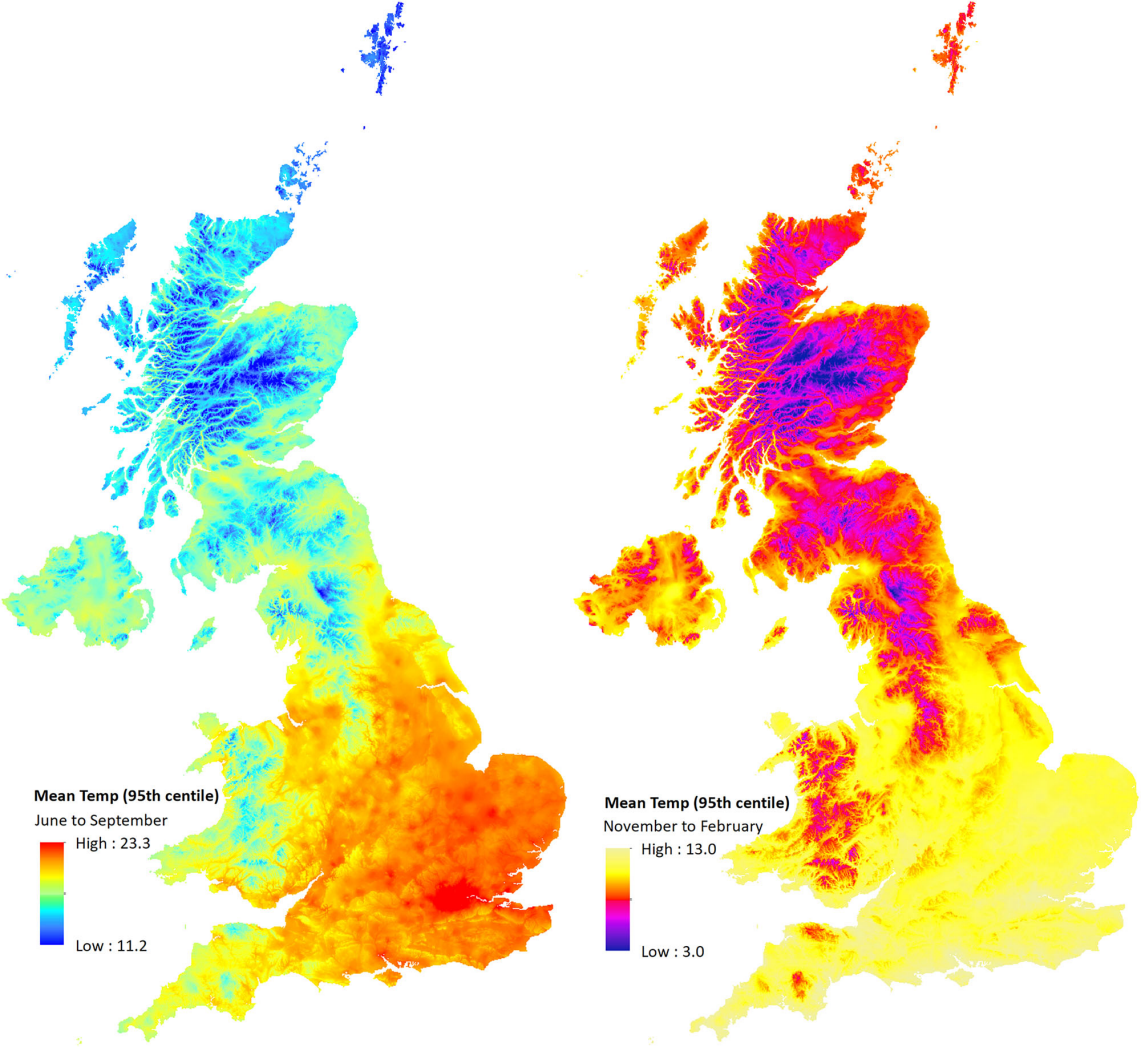
Maps of mean temperature in degree centigrade during warm season (left) and cold season (right) at 1 × 1 Km^2^ resolution over the study period (2006–22) in the UK. Warm season daily mean ambient temperature at the 95^th^ percentile was measured between June and September. Cold season daily mean ambient temperature at the 95^th^ percentile was measured between November and February.

Overall, 709,052 hospitalizations among 259,969 participants were recorded during warm season and 676,686 hospitalizations among 255,604 participants were recorded during cold season between 2006 and 2022 (Supplementary Tables 2 and 3). The mean age at hospitalization during warm season was 66.8 [standard deviation (SD) = 8.6] with 52.3% being women. During cold season, the mean age at hospitalization was 66.5 (SD = 8.5) and 52.2% were women. These participants resided within 25 miles of assessment centres in 22 major cities. Warm season showed higher cause-specific hospitalizations, with cardiovascular disease (*n* = 342,728) being the most common, followed by respiratory disease (*n* = 126,817), mental disorders (*n* = 79,869), renal disease (*n* = 71,751), and heat related illness (*n* = 18,089). Similarly, hospitalizations for cardiovascular disease (*n* = 321,439) were the most common during cold season. High ambient temperature was defined at 20.7 °C for warm (June to September) and 11.1 °C for cold seasons (November to February), representing the 95^th^ percentile of seasonal temperature distributions (Supplementary Table 4). High positive TCN at 95^th^ percentile was 2.9 °C for warm and 2.8 °C for cold seasons. The UK-wide exposure distribution is shown in Fig. 1 and Supplementary Table 4.

**Fig. 2:**
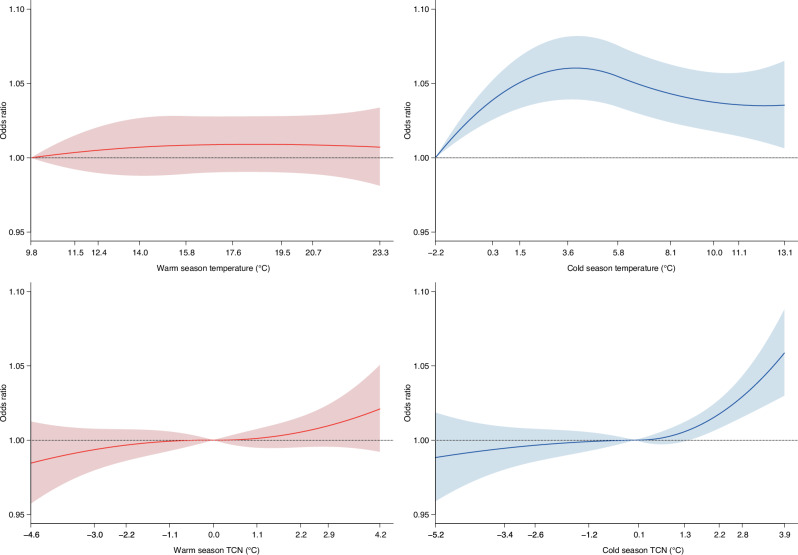
Cumulative exposure-response curves for associations of daily mean ambient temperature and temperature change between neighbouring days (TCN) with hospitalization for any cause. Models adjusted for rainfall and public holiday. The odds ratios (ORs) and 95% confidence intervals (CI) were calculated in reference to the 1^st^ percentile of ambient temperature distribution. For TCN, the ORs and 95% CI were calculated in reference to 0 °C. The number of hospitalizations for any cause is *n* = 709,052 during warm season and *n* = 676,686 during cold season. Solid lines represent ORs of hospitalizations, and shaded areas represent 95% CI. The temperatures shown on the x-axis represent the 1^st^, 5^th^, 10^th^, 25^th^, 50^th^, 75^th^, 90^th^, 95^th^ and 99^th^ percentiles of the temperature distribution.

**Fig. 3:**
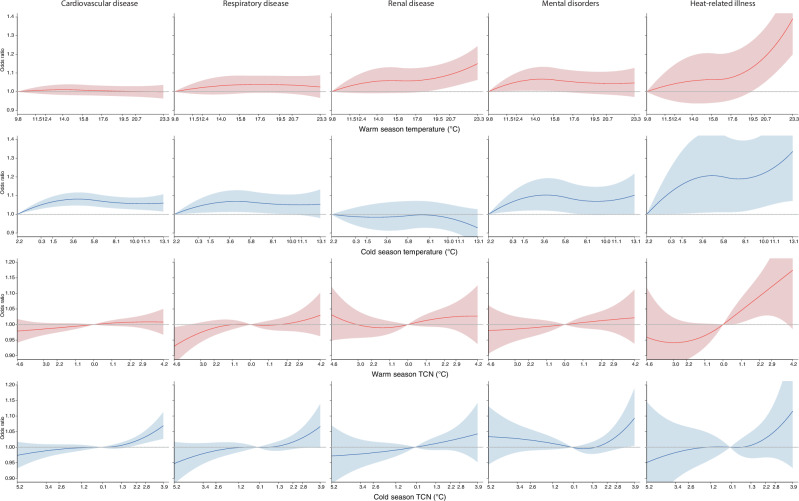
Cumulative exposure-response curves for associations of daily mean ambient temperature and temperature change between neighbouring days (TCN) with cause-specific hospitalizations. Models adjusted for rainfall and public holiday. The odds ratios (ORs) and 95% confidence intervals (CIs) were calculated in reference to the 1st percentile of ambient temperature distribution. For TCN, the ORs and 95% CI were calculated in reference to 0 °C. The number of warm-season hospitalizations is *n* = 342,728, *n* = 126,817, *n* = 71,751, *n* = 79,869, and *n* = 18,089 for cardiovascular disease, respiratory disease, renal disease, mental disorders, and heat-related illness, respectively. The corresponding number of cold-season hospitalizations is 321,439, *n* = 120,763, *n* = 64,060, *n* = 73,438, and *n* = 16,435. Solid lines represent ORs of hospitalizations, and shaded areas represent 95% CI. The temperatures shown on the *x*-axis represent the 1^st^, 5^th^, 10^th^, 25^th^, 50^th^, 75^th^, 90^th^, 95^th^ and 99^th^ percentiles of the temperature distribution.

The associations of high ambient temperature and temperature change between neighbouring days with hospitalizations varied by season. Exposure-response curves are shown in Figs. 2–3. During cold season, high daily ambient temperature corresponding to 95^th^ percentile over cumulated lag 0–3 days was associated with 4% higher odds of hospitalization for any cause (OR = 1.04, 95% CI = 1.01, 1.06), as compared with the referent temperature corresponding to the first percentile of daily ambient temperature at which minimum morbidity was observed (Table 1). This association was most pronounced on the day of diagnosis (lag 0) with reduced effects on the subsequent lag days (Supplementary Fig. 2). This overall association for hospitalization due to any cause was not significant (*p* > 0.05) during warm season. For cause-specific hospitalizations, during warm season, high daily temperature was associated with 9% (OR = 1.09, 95% CI = 1.02, 1.16) higher odds of hospitalizations for renal disease and 18% (OR = 1.18, 95% CI = 1.05, 1.34) higher odds for heat related illness. In the cold season, high daily temperature was also associated with 6% higher odds of hospitalizations for cardiovascular disease (OR = 1.06, 95% CI = 1.02, 1.09), 5% higher odds for respiratory disease (OR = 1.05, 95% CI = 1.00, 1.11), 8% higher odds for mental disorders (OR = 1.08, 95% CI = 1.00, 1.16) and 25% higher odds for heat related illness (OR = 1.25, 95% CI = 1.05, 1.48), in reference to the temperature percentile corresponding to the lowest morbidity.

**Table 1:** Associations of high ambient temperature and positive temperature change between neighbouring days with any cause and cause-specific hospitalizations

	High ambient temperature (95^th^ percentile *vs*. 1^st^ percentile)		Positive temperature change between neighbouring days (TCN) (95^th^ percentile *vs*. 0 °C)	
	Warm season	Cold season	Warm season	Cold season
	OR (95% CI)	OR (95% CI)	OR (95% CI)	OR (95% CI)
Any cause	1.01 (0.99, 1.03)	1.04 (1.01, 1.06)	1.01 (1.00, 1.02)	1.03 (1.02, 1.04)
Cardiovascular disease	1.00 (0.97, 1.03)	1.06 (1.02, 1.09)	1.01 (0.99, 1.03)	1.03 (1.01, 1.05)
Respiratory disease	1.04 (0.99, 1.08)	1.05 (1.00, 1.11)	1.01 (0.98, 1.04)	1.03 (1.00, 1.06)
Renal disease	1.09 (1.02, 1.16)	0.97 (0.90, 1.04)	1.03 (0.98, 1.07)	1.03 (0.98, 1.08)
Mental disorders	1.04 (0.98, 1.11)	1.08 (1.00, 1.16)	1.02 (0.97, 1.06)	1.04 (0.99, 1.08)
Heat related illness	1.18 (1.05, 1.34)	1.25 (1.05, 1.48)	1.12 (1.03, 1.22)	1.05 (0.96, 1.15)

Models adjusted for rainfall and public holiday. The odds ratios (ORs) and 95% confidence intervals (CIs) were calculated for the 95^th^ percentile of ambient temperature distribution in reference to the 1^st^ percentile. For TCN, the ORs and 95% CIs were calculated for the 95^th^ percentile of TCN distribution in reference to 0 °C. Warm season refers to periods between June and September, and cold season refers to periods between November and February for each year.

We observed positive dose-response relationship between TCN (temperature increment) and odds of hospitalization cumulated over lag 0-3 days in both warm and cold seasons (Fig. 2). Positive TCN corresponding to 95^th^ percentile was associated with 1% higher odds of hospitalization for any cause in warm season (OR = 1.01, 95% CI = 1.00, 1.02), and 3% higher odds in cold season (OR = 1.03, 95% CI = 1.02, 1.04), in reference to no temperature difference from previous day (Table 1). Lag-response curve did not show variation in this association between the day of hospitalization (lag 0) and the following lag days during warm season. While, during the cold season, this association was more pronounced on the first day of diagnosis with reduced effects on the subsequent lag days (Supplementary Fig. 3). For cause-specific hospitalizations during warm season, positive TCN, was associated with 12% higher odds of hospitalization for heat related illness (OR = 1.12, 95% CI = 1.03, 1.22) in reference to 0 °C from previous day. During the cold season, positive TCN was associated with 3% higher odds of hospitalizations for cardiovascular disease (OR = 1.03, 95% CI = 1.01, 1.05), and 3% higher odds for respiratory disease (OR = 1.03, 95% CI = 1.00, 1.06).

Our analysis examining effects of moderately high daily temperature defined at the 90^th^ percentile of temperature distribution during the warm season (19.5 °C) found higher odds of hospitalization due to renal disease (OR = 1.07, 95% CI = 1.01, 1.14) and heat related illness (OR = 1.13, 95% CI = 1.00, 1.27). We also observed that moderately positive TCN at 90^th^ percentile of distribution (2.2 °C) was associated with higher odds of hospitalization for any cause (OR = 1.01, 95% CI = 1.00, 1.02) and heat related illness (OR = 1.09, 95% CI = 1.03, 1.16). During cold season, moderately high daily temperature at 90^th^ percentile (10 °C) was associated with higher odds of hospitalizations for any cause (OR = 1.04, 95% CI = 1.02, 1.06), cardiovascular disease (OR = 1.06, 95% CI = 1.03, 1.09), respiratory disease (OR = 1.05, 95% CI = 1.00, 1.11), mental disorders (OR = 1.07, 95% CI = 1.00, 1.15) and heat related illness (OR = 1.22, 95% CI = 1.04, 1.43), relative to the referent temperature corresponding to the lowest morbidity (Supplementary Table 5). Moderately positive TCN during cold season (2.2 °C) was associated with higher odds of hospitalizations for any cause (OR = 1.02, 95% CI = 1.01, 1.03) and cardiovascular disease (OR = 1.02, 95% CI = 1.01, 1.03), in reference to 0 °C change.

Sensitivity analysis showed that the results remained robust to an alternative number of knots for both exposure-response function and lag-response function of temperature (Supplementary Figs. 4–7). The results also remained similar with an alternative modelling of rainfall. We did not observe any systematic difference in the corresponding lag effects models. The results remained robust as in the primary analysis with sensitivity analysis using longer lag period of ten days (Supplementary Table 6). The analysis conducted during-COVID suggested a more pronounced positive association of daily ambient temperature with higher odds of hospitalizations for any cause (OR = 1.11, 95% CI = 1.03, 1.19) and CVD (OR = 1.16, 95% CI = 1.06, 1.28) in the cold season as compared to pre-COVID (any cause: OR = 1.03, 95% CI = 1.01, 1.05; CVD: OR = 1.04, 95% CI = 1.01, 1.08) (Supplementary Tables 7 and 8). Consistently, in warm season, high TCN was associated with relatively higher hospitalizations for mental disorders during COVID (OR = 1.08, 95% CI = 1.00, 1.16). Further analysis including only the first hospitalization record for each participant produced comparable results as in the main analysis (Supplementary Table 9). Further analysis using region-specific temperature percentile thresholds found significantly higher associations of high temperature and TCN with hospitalization for any cause in South East and West (temperature: OR = 1.04, 95% CI = 1.00, 1.08; TCN: OR = 1.03, 95% CI = 1.01, 1.06), and North East and West (temperature: OR = 1.04, 95% CI = 1.00, 1.08; TCN: OR = 1.04, 95% CI = 1.01, 1.06) regions during cold season (Supplementary Data 1).

**Fig. 4:**
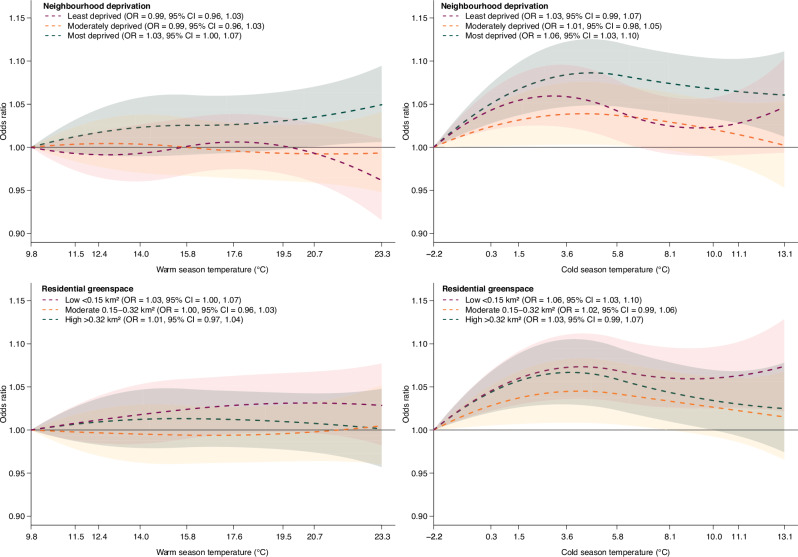
Cumulative exposure-response curves for association between daily mean ambient temperature and hospitalization for any cause by neighbourhood deprivation and residential greenspace. Models adjusted for rainfall and public holiday. In the exposure-response curves, the odds ratios (ORs) and 95% confidence intervals (CIs) were calculated in reference to the 1st percentile of the ambient temperature distribution. Dotted lines represent ORs of hospitalizations, and shaded areas represent 95% CI. The number of warm-season hospitalizations for any cause is *n* = 218,572, *n* = 229,286, and *n* = 260,159 for participants living in the least deprived, moderately deprived and the most deprived neighbourhoods, respectively. The corresponding number of cold-season hospitalization is *n* = 211,320, *n* = 218,838, and *n* = 245,534. The number of warm-season hospitalizations for any cause is *n* = 224,320, *n* = 226,964, and *n* = 228,594 for participants with low, moderate and high levels of residential greenspace coverage, respectively. The corresponding number of cold-season hospitalizations is *n* = 212,410, *n* = 217,798, and *n* = 217,502. The temperatures shown on the x-axis represent the 1^st^, 5^th^, 10^th^, 25^th^, 50^th^, 75^th^, 90^th^, 95^th^ and 99^th^ percentiles of the temperature distribution. In the legends, the ORs and 95% CIs were calculated for the 95^th^ percentile of the ambient temperature distribution in reference to the 1st percentile.

Stratified analysis found heterogeneity in hospitalizations for any cause between population subgroups. During warm season, we found more pronounced association between positive TCN and hospitalization for any cause in participants aged ≥65 years (OR = 1.02, 95% CI = 1.00, 1.04) relative to those <65 years (OR = 0.99, 95% CI = 0.97, 1.01) (*P*-value for heterogeneity = 0.033) (Supplementary Data 2). In the stratified analysis by sex, male (temperature: OR = 1.04, 95% CI = 1.01, 1.07; TCN: OR = 1.04, 95% CI = 1.02, 1.06) showed slightly more pronounced associations of high temperature and positive TCN during cold season with odds of hospitalization from any cause as compared with female (temperature: OR = 1.03, 95% CI = 1.00, 1.06; TCN: OR = 1.02, 95% CI = 1.00, 1.04), though the heterogeneity between subgroups remained insignificant (Supplementary Data 3). We also found more pronounced associations between high temperature and hospitalization for any cause in subgroups residing in the most deprived (warm: OR = 1.03, 95% CI = 1.00, 1.07; cold: OR = 1.06, 95% CI = 1.03, 1.10) and with the lowest residential greenspace (<0.27 Km^2^) (warm: OR = 1.03, 95% CI = 1.00, 1.07; cold: OR = 1.06, 95% CI = 1.03, 1.10) exposure tertiles during both warm and cold seasons. There were no significant differences between groups in the associations (Fig. 4). The association between cold season TCN and hospitalization for any cause was also more pronounced among subgroups residing in the most deprived neighbourhoods (OR = 1.05, 95% CI = 1.03, 1.08) as compared with the least deprived (OR = 1.01, 95% CI = 0.99, 1.03) and moderately deprived (OR = 1.02, 95% CI = 1.00, 1.05) subgroups (*P*-value for heterogeneity = 0.031). Similarly, the association between cold season TCN and hospitalization for any cause was more pronounced among subgroups residing in neigbourhoods of the highest residential unit density (OR = 1.05, 95% CI = 1.03, 1.08) relative those of low (OR = 1.00, 95% CI = 0.98, 1.03) and moderate densities (OR = 1.03, 95% CI = 1.00, 1.05) (*P*-value for heterogeneity=0.013). For natural greenspace, more pronounced associations of high temperature (warm: OR = 1.02, 95% CI = 0.98, 1.05; cold: OR = 1.05, 95% CI = 1.01, 1.09) and positive TCN (warm: OR = 1.03, 95% CI = 1.00, 1.05; cold: OR = 1.04, 95% CI = 1.01, 1.06) during both seasons were identified in subgroups residing in neighbourhoods with the least natural greenspace, although no significant heterogeneity was identified between subgroups. For physical health, the associations of high temperature and TCN with hospitalization for any cause during cold season was found to be more pronounced among participants with pre-frail or fail conditions (temperature: OR = 1.04, 95% CI = 1.00, 1.07; TCN: OR = 1.04, 95% CI = 1.02, 1.06), and a low-level of physical activity (temperature: OR = 1.05, 95% CI = 1.00, 1.10; TCN: OR = 1.04, 95% CI = 1.01, 1.07), with insignificant heterogeneity between subgroups. In the subgroup analysis by climatic regions of UK, we found that high temperature (OR = 1.11, 95% CI = 1.01, 1.22) and TCN (OR = 1.06, 95% CI = 1.00, 1.13) in warm season were associated with relatively higher hospitalization for any cause in Scotland. During cold season, the associations of high temperature and TCN with hospitalization for any cause remained in South East and West (temperature: OR = 1.05, 95% CI = 1.00, 1.09; TCN: OR = 1.03, 95% CI = 1.01, 1.06), and North East and West region (temperature: OR = 1.04, 95% CI = 1.00, 1.08; TCN: OR = 1.04, 95% CI = 1.02, 1.07).

## Discussion

Using 1.38 million hospitalization records from a nationwide cohort, we found a positive association between ambient temperature and all-cause and cause-specific hospitalizations, which varied by season in temperate conditions of UK. During warm season, high temperature was associated with higher odds of hospitalizations for renal and heat related illness. During cold season, high temperature was associated with higher odds of hospitalizations for any cause, and for cardiovascular disease, respiratory disease, mental disorders and heat related illness. Consistently, positive TCN was associated with higher hospitalizations from any cause and heat-related illness during warm season and hospitalizations from any cause, CVD, and respiratory disease during cold season. Our results remained robust to a range of sensitivity tests including alternative number of key parameters in the cross-basis function and rainfall modelling. Of note, were the findings of stratified analysis, showing more pronounced associations between ambient temperature and overall hospitalization among patient subgroups residing in the most deprived neighbourhoods and those with least greenspace coverage during both warm and cold seasons.

Nationwide cohort studies employing case-crossover design to examine associations between ambient temperature and TCN on hospitalizations have been very scarce. It is less likely that our evidence can be directly compared with prior studies with substantial heterogeneity in the spatial resolution of exposure measurement, exposure metrics, outcome assessments, climatic zone, study period and season, population and statistical approach. Majority of the studies have focused analysis on the summer season^30,35^ and measured temperature exposure relying on few nearest meteorological monitoring stations^35,51,52^ or at more aggregated spatial unit such as county^30^ or city^53^, that are less likely to capture spatial heterogeneity.

Our findings that high temperature (95^th^ percentile) was positively associated with hospitalizations for renal disease and heat related illness during warm season corroborate past evidence^30,32,53–56^. Previously, a time-series study in Central Australia found that a high daily temperature at 31 °C cumulated over lag 0–1 days was associated with a higher risk of hospitalization for any chronic kidney disease (RR = 1.27), as compared with the minimum risk temperature at 5.1 °C^54^. Another time-series analysis from Israel reported that the number of emergency-room visits for renal colic increased during summertime (July to October) and peaked in August^55^. Among the evidence for case-crossover design, a study in South Korea reported a 1.5% higher odds of emergency department visits for acute kidney injury for each 1 °C increase in warm season temperature^53^. A Brazilian study employing similar design reported 0.9% higher risk of hospitalization for renal disease for every 1 °C increase in daily mean temperature^32^. Consistently, another case-crossover study in the US showed that extreme temperature (34.4 °C) during warm season over lag 0–5 days was associated with 66.3% and 30.4% excess relative risks of emergency department visits for heat related illness and renal disease, respectively^30^. A matched case-control study from Ontario, Canada found that heat periods were associated with 11% higher odds of acute kidney injury hospitalization^56^. It has been suggested that high temperature increases core body temperature, resulting in heat stress and thermoregulatory dysfunction^57^. Heat-induced sweating may cause dehydration and salt depletion, leading to a higher risk of heat related illnesses^58^. It is plausible that short-term high temperature exposure may lead to sudden increment in serum creatinine levels [a proxy of estimated glomerular filtration (eGRF)]^59^, rapid alteration in blood circulation^60^, or abnormality of plasma 25-hydroxyvitamin D^61^, which are all risk factors of renal disease.

During cold season, we found that high ambient temperature was associated with higher odds of hospitalizations for cardiovascular disease and respiratory disease. The results also remained consistent in our analysis examining associations with positive TCN. Previously, a nationwide Swedish coronary care unit registry study using time series design, reported a positive association between higher air temperature and daily counts of hospitalization due to myocardial infarction (IRR = 1.025) during the coldest season over January to March^62^. Consistently, a study in Hong Kong reported significantly higher excess relative risk of emergency hospitalization for heart failure associated with 1 °C increment of diurnal temperature range during cool season relative to warm season^63^. We did not observe significant association between cardiovascular disease and ambient temperature and TCN during the warm season, as in another largescale US study^30^. The observed positive association between higher winter temperature and its increment and hospitalization due to respiratory disease may plausibly be linked to shifting seasonal patterns, especially shorter and milder winters attributed to global warming induced perturbations in seasonal clock^4,64^. Respiratory viruses are highly seasonal in temperate regions with late winter to early spring peaks resulting in higher caseloads in shortening winters with milder temperatures. The positive association between positive TCN and hospitalizations for mental disorders during cold season supplement prior evidence showing the potential adverse psychological effects of milder winter temperatures. A recent US-wide case-crossover study of older adults aged ≥65 years enrolled in the Medicare programme found that every 5 °C increment in short-term exposure to temperature during cold season measures at a spatial resolution of 12 × 12 Km^2^ was associated with higher relative risk of hospital admission for depression (3.7%), schizophrenia (3.0%) and for bipolar disorder (3.5%)^33^, while the associations were not significant during warm season (*p* > 0.05), consistent with our study. Another Californian study using time series analysis found 5.3% higher risks of emergency room visits for mental disorders for increased temperatures during cold season and 4.8% during warm season^65^.

Our findings of positive associations between high temperature and hospitalization during cold season may be attributed to underlying health-seeking behaviours, medication intake, and established heating systems during extremely cold season in temperate region. Extreme cold winter days may disincentivise hospital visits leading to postponement or delayed clinical consultations and absence of timely treatment, potentially resulting in lagged effects only observed at milder temperatures. Paradoxically, susceptible patients with serious conditions could have been died before hospital admission. This could be supported by the growing evidence of higher mortality risks during extreme cold season^66^. In our study, the lack of evidence of positive association between extreme cold temperature and hospitalizations may plausibly be indicative of the well-established heating and insulation systems in household settings throughout the UK^67^. The null results during summers may be reflective of increase in net recreational physical activity during warmer seasons^68^ and its protective effects on cardiovascular^69^ and mental health^70^.

Our subgroup analysis by COVID-19 period identified more pronounced positive associations of temperature with hospitalizations for any cause and CVD in cold season during the COVID as compared to pre-COVID. These findings may have captured the elevated adverse health effects of indoor living and work environments during COVID-induced public health emergency^71^. The positive association between hospitalization for mental disorders and change in temperature in warm season during COVID period may plausibly indicate the exacerbated risks associated with restrictive mobility and work from home arrangements^72^. It has been suggested that the disruptive societal change brought in by introductions of stay-at-home mandate, social distancing and restrictive use of public recreational facilities maybe detrimental to mental health^73^. These conditions may have amplified the impact of heat on mental health, especially among those residing in high density urban areas with limited living space and without cooling devices at home, resulting in a higher risk of hospitalization.

Analysis by climatic regions identified the highest risk of hospitalization for any cause in Scotland during warm season, and in South East and West, and North East and West regions during the cold season. Further sensitivity analysis based on region-specific temperature percentiles found that high temperature and TCN were associated with higher hospitalization for any cause in South East and West, and North East and West regions during cold season. Our findings corroborate with existing literatures showing a higher risk of hospitalization attributable to warm season high temperature among residents living in the north relative to the south^30,32^, and higher hospitalizations in cold season among residents residing in warmer regions^33^. These spatial effect variations may reflect the differential adaptation capacities corresponding to the heterogeneity in physiological responses to high and low temperature extremes among residents acclimatized to different environments of UK^74^. Such nuanced evidences may provide insights in to spatial climate adaptation policies.

The pronounced positive association between temperature and overall hospitalization among patient subgroups residing in the most deprived neighbourhoods and with low residential greenspace coverage during both warm and cold seasons may have significant implications on planning and design of climate-resilient cities. It is plausible that deprived neighbourhoods may be a proxy of poor physical environment, poor design and inherent structural inequities in access to resources. These factors may exacerbate the intensity and sensitivity to climate risks reducing capacity for climate mitigation and adaptation. At an individual level, the subgroup with low socio-economic status may potentially have lower financial capacity and awareness of climatic preparedness, and as such, are vulnerable to the detrimental health effects of extreme temperature^75,76^. Population residing in urban neighbourhoods with low greenspace coverage may intensify the detrimental health effects of elevated temperature in absence of the nature-based cooling solution^77,78^. These findings are consistent with existing evidence showing the adverse effects of climate change on socioeconomically vulnerable population^27,28^ and those in absence of nature-based health risk-proofing^29^. The observed pronounced effects among subgroups living in neighbourhoods of higher residential unit density during cold season may have been induced by heat island effect. High density environments are essentially agglomerations of buildings and impervious spaces that perturb the radiation budget causing canopy layer heat island effects^79^ elevating local temperatures. We also identified more pronounced association between positive TCN and overall hospitalization among patients aged ≥65 years (OR = 1.02, 95% CI = 1.00, 1.04) relative to those <65 years (OR = 0.99, 95% CI = 0.97, 1.01) during warm season. Prior evidence has shown narrower thermal acceptability range^80^ and greater physiological response (higher blood pressure) in older adults relative to younger counterparts during summertime^81^. Additionally, population with underlying comorbidities such as diabetes^25^ and hypertensive disease^82^ are likely to be susceptible to extreme temperature exposures due to potential thermoregulatory dysfunctions that involve autonomic nervous and circulatory systems, leading to a higher risk of hospitalization. Additionally, other social and behavioural traits among older adults, such as living alone, reduced mobility, limited financial resources for adaptation, and declined behavioural response to thermal stress may all exacerbate their vulnerability to temperature extremes^24^. These results reflect the interplay of greenness, density, deprivation and population age in the socio-spatial distribution of climate-attributed health vulnerabilities in cities and highlight the importance of multi-stakeholder urban planning^83,84^ to enable adaptation at population level, especially among older adults with deteriorated physical and cognitive functioning as well as lower adaptability to climate change.

Among the strengths of the study, to our knowledge this is the first UK-wide largescale cohort study using the time-stratified case-crossover design to examine associations between ambient temperature and hospitalization. Such a design was able to inherently address the issue of confounding attributable to time-invariant factors, and seasonal and long-term trends. Second, our study generated dose-response curves with ambient temperature for all-cause and various key cause-specific hospitalizations providing deeper insights in to the health effects of global warming. We additionally estimated risks associated with temperature change employing a more nuanced indicator of temperature difference between neighbouring days. Our models reported consistent results as with mean temperature, and thus extended prior knowledge of the health impacts of global warming. Third, leveraging a well-characterized cohort, our study used a high resolution 1 × 1 Km^2^ gridded estimates of mean ambient temperature and linked them to geocoded home address coordinates of UK Biobank participants. Several prior studies directly used data derived from nearest meteorological stations to represent spatially heterogeneous study areas^34,35^, potentially resulting in potential exposure misclassification and biasing results to either side. Lastly, reliance on a well-characterized cohort with individual-level data on traits such as physical activity, frailty and environmental exposures such as density, green coverage and deprivation enables us to reliably examine effect modifications by these variables. Such evidence is important for climate change focused on planning and design towards creation of resilient communities and cities.

Our study had several limitations. First, the UK Biobank cohort of middle and older aged participants had low response rate and is susceptible to healthy volunteer bias^85^, and as such, the hospitalization rates may not be generalizable to normal UK population, including population sub-groups such as adolescents and children and other vulnerable populations. Second, although we employed time-stratified case-crossover design, residual confounding attributable to factors that may vary at short intervals (lag periods), such as other climatic factors and air pollution cannot be entirely ruled out^42^. Specifically, the interaction between temperature extremes and short-term air pollution exposures on hospitalizations warrants further explorations^86,87^. For example, ambient temperature has been found to be linked with blood pressure via the blood vessels constriction and dilation pathways^88^, whereas fine particulate matter PM_2.5_ can induce autonomic nervous system imbalance^89^, and hence the potential synergistic effect of temperature extremes and air pollution on the risk of cardiovascular hospitalization requires further explorations. Typically, urban areas are prone to heat island effect and poor ground-level ventilation. As such, extreme high temperatures may potentially elevate ground-level air pollutants such as ozone, inducing higher risks of asthma and respiratory hospitalizations^90^. Third, although temperature exposures were modelled at fine resolution of 1 × 1 Km^2^ grids, we could not account for personalized exposure and indoor thermal comfort in our models which could have introduced some degree of misclassification. Exposure misclassification may arise from daily mean temperature exposure calculations based on averaging the minimum and maximum temperature values due to an absence of consistent data on hourly mean temperature accumulated over 24 h of a day from all monitoring stations over the study period. Nonetheless, such misclassification is likely to be non-differential. Fourth, our results should be interpreted with caution owing to the fact that UK Biobank population is predominantly urban, with approximately 86% of the participants resided in urban areas. This implies that our findings may not be generalizable to those living in suburban or remote rural areas. Fifth, while our study showed increased respiratory hospitalizations for higher winter temperatures, we were unable to adjust for the potential confounding by circulating infectious disease outbreaks such as influenza that often follows seasonal transmission patterns^91^. Sixth, our study examined the associations between temperature extremes and in-patient hospitalizations, which would have included both emergency and pre-planned hospitalizations. It must be acknowledged that extreme climatic events including extreme temperatures pose stress on the healthcare system by raising demand for services and increasing potential for their disruption. Health service utilization depends on their ease of accessibility, available transport services, urban density and other factors such as weather-related service disruptions. In urban areas with higher provisioning of healthcare services and transportation, studies have shown a positive correlation between extreme climatic events and utilization patterns^92^. In relatively remote semi-urban and rural areas with low density of facilities, the utilization rate may plausibly be low, owing to higher chances of service disruption, difficulty in accessing them and perceived risks associated with travelling during extreme climatic events. Future studies should factor in health service utilization patterns during extreme temperature events as well as account for accessibility and density of healthcare services and their probability of disruption. Lastly, a time-stratified approach may imply that some referent days are selected after the case day when an individual already experienced the event, which may result in some residual bias. For example, an individual who had been hospitalized could either be treated or more vulnerable to heat or cold given deteriorated health conditions following an event, which may bias the estimates to either side. Nonetheless, compared to other referent sampling strategies (such as fixed unidirectional retrospective approach that selects referent days only before the case day, and symmetric bi-directional approach that selects referent days at a fixed interval both before and after the case day), the time-stratified approach has been found to provide the least-biased estimates^93,94^. We reviewed existing referent selection strategies, by searching in PubMed and Google Scholar (using keywords “temperature”, “hospitalization” and “case-crossover”) for studies of case-crossover design published in the recent five years. We found that 82% of the identified studies selected referent days following a time-stratified approach (Supplementary Data 4). A time-stratified approach partitions time into disjoint strata and ensures that cases and controls are closely aligned in time, thereby being able to avoid overlap bias and account for long term time trends and seasonality.

## Conclusion

Among middle and older aged adults in a UK-wide cohort, high ambient temperature was associated with higher odds of hospitalizations for renal and heat related illness during warm season, and higher odds of hospitalizations for any cause, cardiovascular disease, respiratory disease, mental disorders and heat related illness during cold season. A more pronounced association between warm season high temperature and hospitalization for any cause was identified in subgroups residing in the most deprived neighbourhoods and with low greenspace coverage. Our findings add to the body of evidence on underlying population vulnerability to extreme temperature and its variability. These findings have important implications for governments, local community planners, public health practitioners and the general public to design and operationalize actionable climate risk mitigation and adaptation strategies for individual and urban resilience. Individual-level strategies can be customized to protect residents from extreme temperature accounting for lifestyle, behaviors and underlying health conditions. Planning and designing of neighborhoods and cities with adequate provisions of climate-resistant infrastructures can strengthen urban resilience. These multi-level strategies, when applied holistically, can have the potential to minimize health costs attributable to climate extremes.

## Supplementary information

Supplementary Information

Description of Additional Supplementary Data

Supplementary Data 1

Supplementary Data 2

Supplementary Data 3

Supplementary Data 4

Reporting summary

## Data Availability

Source data for the figures can be found in the main Table and Supplementary Data Files. Specifically, the source data that support Figs. 2 and 3 is in Table 1, and the source data that support Fig. 4 is in Supplementary Data Files 2-3. UK Biobank data including the linked climatic exposure metrics are available from the UK Biobank at https://www.ukbiobank.ac.uk/ for researchers who meet the criteria for access to de-identified data. The daily mean temperature and rainfall data were sourced from the UK Met Office’s HadUK-Grid dataset at https://www.metoffice.gov.uk/research/climate/maps-and-data/data/haduk-grid/datasets and linked to the UK Biobank.

## Code availability

The analysis was conducted using custom-made scripts in R v.4.4.2. The study is part of a project approved by UK Biobank under a restricted Material Transfer Agreement and hence computer codes are not publicly available.
