# Supplementary material for: Ambient temperature and the variability between neighbouring days impacts in-patient hospitalizations in the United Kingdom
Source: Commun Med (Lond). 2026 Jan 26;6:90. doi: 10.1038/s43856-025-01355-y (PMC12881400; doi:10.1038/s43856-025-01355-y)
Supplement: Supplementary file 1 — Supplementary Information [file 43856_2025_1355_MOESM1_ESM.pdf]

Supplementary Information

Ambient temperature and the variability between neighbouring days impacts in-patient hospitalizations in the United Kingdom

Ka Yan Lai[1,2,3*], Chris Webster[1,3,4], John Gallacher[5], Chinmoy Sarkar[1,2,3,5]

[1]Healthy High Density Cities Lab, HKUrbanLab, The University of Hong Kong, Hong Kong Special Administrative Region, China
[2]Institute for Climate and Carbon Neutrality, The University of Hong Kong, Hong Kong Special Administrative Region, China
[3]Department of Urban Planning & Design, Faculty of Architecture, The University of Hong Kong, Hong Kong Special Administrative Region, China
[4]Urban Systems Institute, The University of Hong Kong, Hong Kong Special Administrative Region, China
[5]Department of Psychiatry, University of Oxford, Oxford, United Kingdom

**Correspondence*:
Healthy High Density Cities Lab, HKUrbanLab, The University of Hong Kong, Knowles Building, Pokfulam Road, Pokfulam, Hong Kong Special Administrative Region, China.

Supplementary Method

- Age was calculated from the date of birth to the date of hospitalization.

- Neighbourhood deprivation was measured by the Townsend deprivation index, a composite measure from four components; unemployment rate, overcrowding, non-car ownership and non-home ownership measured for each output areas (census aggregate) and derived from the 2011 national census data. The output area is the lowest geographical level of aggregated census data. Each output area is equivalent to a minimum of 40 households for England and Wales, and 20 in Scotland. Participants were assigned a score linked via their postcode of residence.

- Frailty was assessed at baseline from five phenotypes; weight loss, exhaustion, low grip strength, low physical activity and slow gait speed and coded as non-frail, pre-frail/frail[1,2].

  (1) Weight loss was derived from the questionnaire on weight change, 'Compared with one year ago, has your weight changed?', with the option to select from: 'No - weigh about the same', 'Yes - gained weight', 'Yes - lost weight', 'Do not know', 'Prefer not to answer'.

  (2) Low gait speed was derived from the questionnaire, 'How would you describe your usual walking pace?', with the option of selecting one from: 'Slow pace', 'Steady average pace', 'Brisk pace', 'None of the above', and 'Prefer not to answer' which was recoded as a binary variable.

  (3) Grip strength was measured with the use of a Jamar J00105 hydraulic hand dynamometer. Participants were asked to sit upright in a chair and squeeze the handle of the dynamometer as strongly as they can with the use of their left hand for approximately 3 seconds. The grip strength measurement was then repeated by using their right hand. The higher value of the two grip strength measurements was used in the analysis. Low grip strength was defined based on sex and body-mass index adjusted thresholds (Men: ≤29 for BMI≤24, ≤30 for BMI≥24.1 and ≤28, ≤32 for BMI>28; Women: ≤17 for BMI≤23, ≤17.3 for BMI≥23.1 and ≤26, ≤18 for BMI≥26.1 and ≤29, ≤21 for BMI>29).

  (4) Exhaustion was evaluated based on the question 'Over the past two weeks, how often have you felt tired or had little energy?' with the option to select from 'not at all', 'Several days', 'More than half the days', 'Nearly every day', 'Do not know', 'Prefer not to answer'. Participants who answered 'More than half the days' or 'Nearly every day' met the criteria of exhaustion.

  (5) Low physical activity was evaluated from the question, 'In the last 4 weeks did you spend any time doing the following? (You can select more than one answer)'. The responses were classified as none (no physical activity), low (light DIY activities such as pruning, watering the lawn), medium (including heavy DIY activities (such as weeding, lawn mowing, carpentry, digging), walking for pleasure or other exercises) and high (strenuous sports). Participants who conducted non or light physical activity over the past 4 weeks met the criteria of low physical activity. Participants who did not meet any of the five frailty criteria were defined as not frail, while meeting any one or two were classified as pre-frail and three or more as frail.

- The residential density metrics employed in this study were obtained from the UK Biobank Urban Morphometric platform[3], a built environment exposure database we had developed for measuring urban exposures. We used the Ordnance Survey GB's AddressBase Premium dataset

comprising 36 million valid address point features with 550 different land-use classifications to measure land-use densities around the residential catchments of cohort participants' geocoded residences. The data was sourced from local authorities and Royal Mail. We also employed OSGB's street network database, the Integrated Transport Network (ITN) layer comprising street centerline links. The participants' residential addresses were first geocoded and neighbourhoods comprising street catchments of 1.0 kilometers were delineated.
Residential density was measured as:

$$ResidentialD_i^{1\ Km} = \frac{n_1RD + n_2RD02 + n_3RD03 + n_4RD04 + n_5RD06}{A} \quad \text{………… (1)}$$

where $ResidentialD_i^{1\ Km}$ is the residential density for a cohort participant 'i' within an 1 Km street catchment neighbourhood; n_1, n_2, n_3, n_4, and n_5 are the number of housing units within the various housing typologies, namely, residential-unclassified (*RD*), detached (*RD02*), semi-detached (*RD03*), terraced (*RD04*), and self-contained flats (*RD06*) respectively and A is area under 1 Km residential street network catchment.

- Residential greenspace was measured as area in Km^2 within 1000-metre circular buffers from each cohort participant's home. The greenspace dataset was derived from the England Green and Blue Infrastructure Database and Scottish datasets as before[4]. In the present study, residential greenspace is calculated by the sum of areas of natural greenspace, urban parks, allotments and cemeteries in square metres.

Supplementary Table 1. International Classification of Diseases (ICD) codes used in the UK Biobank for identification of cases of hospitalizations in the UK Biobank.

Disease	ICD-10 codes	Category
Any cause	A00-Y98	
Cardiovascular disease	I00-I99	Diseases of the circulatory system
Respiratory disease	J30-J98, excluding J69, J96	
	J30-J39	Other diseases of upper respiratory tract
	J40-J47	Chronic lower respiratory diseases
	J60-J70, excluding J69	Lung diseases due to external agents
	J80-J84	Other respiratory diseases principally affecting the interstitium
	J85-J86	Suppurative and necrotic conditions of lower respiratory tract
	J90-J94	Other diseases of pleura
	J95-J98, excluding J96	Other diseases of the respiratory system
Renal disease	N00-N05, N08, N17-N19, N25-N27	
	N00	Acute nephritic syndrome
	N01	Rapidly progressive nephritic syndrome
	N02	Recurrent and persistent hematuria
	N03	Chronic nephritic syndrome
	N04	Nephrotic syndrome
	N05	Unspecified nephritic syndrome
	N08	Glomerular disorders in diseases classified elsewhere
	N17	Acute kidney failure
	N18	Chronic kidney disease (CKD)
	N19	Unspecified kidney failure
	N25	Disorders resulting from impaired renal tubular function
	N26	Unspecified contracted kidney
	N27	Small kidney of unknown cause
Mental health disorder	F01-F99	Mental, behavioral and neurodevelopmental disorders
Heat-related disorders	E86-E87, T67, X30	
	E86	Volume depletion
	E87	Other disorders of fluid, electrolyte and acid-base balance
	T67	Effects of heat and light
	X30	Exposure to excessive natural heat

Supplementary Table 2. Characteristics of the study population during warm season.

	Any cause	Cardiovascular disease	Respiratory disease	Renal disease	Mental disorders	Heat related illness
Number of cases	709,052	342,728	126,817	71,751	79,869	18,089
Age at hospital visits	66.8±8.6	69.4±7.7	68.3±8.4	69.9±8.4	66.7±9.1	71.1±7.7
Sex						
Female	370,533 (52.3)	154,298 (45.0)	68,089 (53.7)	29,711 (41.4)	42,126 (52.7)	8,768 (48.5)
Male	338,519 (47.7)	188,430 (55.0)	58,728 (46.3)	42,040 (58.6)	37,743 (47.3)	9,321 (51.5)
Neighbourhood deprivation						
Least deprived (tertile 1)	218,572 (30.8)	100,843 (29.4)	33,031 (26.1)	17,498 (24.4)	18,014 (22.6)	4,841 (26.8)
Moderately deprived (tertile 2)	229,286 (32.3)	110,538 (32.3)	37,400 (29.5)	21,577 (30.1)	22,037 (27.6)	5,523 (30.5)
Most deprived (tertile 3)	260,159 (36.7)	130,930 (38.2)	56,262 (44.4)	32,051 (44.7)	39,717 (49.7)	7,703 (42.6)
Missing	1,035 (0.2)	417 (0.1)	124 (0.1)	625 (0.9)	101 (0.1)	22 (0.1)
Frailty						
Non-frail	216,421 (30.5)	91,872 (26.8)	30,155 (23.8)	14,285 (19.9)	18,121 (22.7)	4,462 (24.7)
Pre-frail/Frail	301,850 (42.6)	154,193 (45.0)	57,904 (45.7)	35,080 (48.9)	36,953 (46.3)	8,026 (44.4)
Missing	190,781 (26.9)	96,663 (28.2)	38,758 (30.6)	22,386 (31.2)	24,795 (31.0)	5,601 (31.0)
Physical activity						
Normal or high	420,176 (59.3)	195,392 (57.0)	68,692 (54.2)	34,834 (48.6)	43,307 (54.2)	9,993 (55.2)
Low	130,309 (18.4)	66,988 (19.6)	25,671 (20.2)	19,925 (27.8)	15,926 (19.9)	3,565 (19.7)
Missing	158,567 (22.4)	80,348 (23.4)	32,454 (25.6)	16,992 (23.7)	20,636 (25.8)	4,531 (25.1)
Residential density (unit/Km^2)						
Low (tertile 1; <1,447)	212,854 (30.0)	99,925 (29.2)	34,086 (26.9)	16,755 (23.4)	18,855 (23.6)	4,723 (26.1)
Moderate (tertile 2; 1,447 to 2,094)	233,044 (32.9)	113,335 (33.1)	41,113 (32.4)	23,231 (32.4)	25,644 (32.1)	5,849 (32.3)
High (tertile 3; >2,094)	236,215 (33.3)	116,263 (33.9)	46,446 (36.6)	29,050 (40.5)	31,388 (39.3)	6,701 (37.0)
Missing	26,939 (3.8)	13,205 (3.9)	5,172 (4.1)	2,715 (3.8)	3,982 (5.0)	816 (4.5)
Residential greenspace (Km^2)						
Low (tertile 1; <0.27)	224,320 (31.6)	107,698 (31.4)	39,063 (30.8)	22,871 (31.9)	25,286 (31.7)	5,923 (32.7)
Moderate (tertile 2; 0.27 to 0.49)	226,964 (32.0)	110,747 (32.3)	41,498 (32.7)	23,663 (33.0)	26,039 (32.6)	5,863 (32.4)
High (tertile 3; >0.49)	228,594 (32.2)	109,726 (32.0)	41,082 (32.4)	23,880 (33.3)	25,685 (32.2)	5,792 (32.0)
Missing	29,174 (4.1)	14,557 (4.3)	5,174 (4.1)	1,337 (1.9)	2,859 (3.6)	511 (2.8)
Natural greenspace (Km^2)						
Low (tertile 1; <0.15)	228,738 (32.3)	107,682 (31.4)	40,172 (31.7)	25,448 (35.5)	26,742 (33.5)	6,202 (34.3)
Moderate (tertile 2; 0.15 to 0.32)	226,757 (32.0)	112,253 (32.8)	41,702 (32.9)	23,360 (32.6)	26,370 (33.0)	5,876 (32.5)
High (tertile 3; >0.32)	224,383 (31.7)	108,236 (31.6)	39,769 (31.4)	21,606 (30.1)	23,898 (29.9)	5,500 (30.4)
Missing	29,174 (4.1)	14,557 (4.3)	5,174 (4.1)	1,337 (1.9)	2,859 (3.6)	511 (2.8)

Data are presented as mean ± standard deviation or as number (%).

Supplementary Table 3. Characteristics of the study population during cold season.

	Any cause	Cardiovascular disease	Respiratory disease	Renal disease	Mental disorders	Heat related illness
Number of cases	676,686	321,439	120,763	64,060	73,438	16,435
Age at hospital visits	66.5±8.5	69.1±7.6	68.0±8.3	69.6±8.3	66.5±8.9	70.7±7.6
Sex						
Female	352,893 (52.2)	144,565 (45.0)	65,008 (53.8)	26,543 (41.4)	38,262 (52.1)	7,888 (48.0)
Male	323,793 (47.9)	176,874 (55.0)	55,755 (46.2)	37,517 (58.6)	35,176 (47.9)	8,547 (52.0)
Neighbourhood deprivation						
Least deprived (tertile 1)	211,320 (31.2)	96,435 (30.0)	30,981 (25.7)	15,578 (24.3)	16,655 (22.7)	4,309 (26.2)
Moderately deprived (tertile 2)	218,838 (32.3)	103,577 (32.2)	36,157 (29.9)	19,627 (30.6)	20,394 (27.8)	5,077 (30.9)
Most deprived (tertile 3)	245,534 (36.3)	121,023 (37.7)	53,543 (44.3)	28,343 (44.2)	36,283 (49.4)	7,031 (42.8)
Missing	994 (0.2)	404 (0.1)	82 (0.1)	512 (0.8)	106 (0.1)	18 (0.1)
Frailty						
Non-frail	208,900 (30.9)	87,439 (27.2)	28,623 (23.7)	12,195 (19.0)	16,769 (22.8)	3,856 (23.5)
Pre-frail/Frail	286,785 (42.4)	144,256 (44.9)	55,476 (45.9)	31,699 (49.5)	33,619 (45.8)	7,533 (45.8)
Missing	181,001 (26.8)	89,744 (27.9)	36,664 (30.4)	20,166 (31.5)	23,050 (31.4)	5,046 (30.7)
Physical activity						
Normal or high	401,954 (59.4)	183,844 (57.2)	65,472 (54.2)	30,607 (47.8)	39,774 (54.2)	8,892 (54.1)
Low	124,455 (18.4)	62,931 (19.6)	24,698 (20.5)	18,142 (28.3)	14,578 (19.9)	3,460 (21.1)
Missing	150,277 (22.2)	74,664 (23.2)	30,593 (25.3)	15,311 (23.9)	19,086 (26.0)	4,083 (24.8)
Residential density (unit/Km^2)						
Low (tertile 1; <1,447)	206,130 (30.5)	95,287 (29.6)	32,342 (26.8)	14,861 (23.2)	17,529 (23.9)	4,233 (25.8)
Moderate (tertile 2; 1,447 to 2,094)	221,220 (32.7)	106,557 (33.2)	39,184 (32.5)	20,826 (32.5)	23,340 (31.8)	5,293 (32.2)
High (tertile 3; >2,094)	223,814 (33.1)	107,371 (33.4)	44,355 (36.7)	26,020 (40.6)	28,896 (39.4)	6,211 (37.8)
Missing	25,522 (3.8)	12,224 (3.8)	4,882 (4.0)	2,353 (3.7)	3,673 (5.0)	698 (4.3)
Residential greenspace (Km^2)						
Low (tertile 1; <0.27)	212,410 (31.4)	100,948 (31.4)	37,091 (30.7)	20,310 (31.7)	22,748 (31.0)	5,237 (31.9)
Moderate (tertile 2; 0.27 to 0.49)	217,798 (32.2)	104,184 (32.4)	39,657 (32.8)	20,996 (32.8)	24,425 (33.3)	5,427 (33.0)
High (tertile 3; >0.49)	217,502 (32.1)	102,071 (31.8)	38,719 (32.1)	21,477 (33.5)	23,311 (31.7)	5,227 (31.8)
Missing	28,976 (4.3)	14,236 (4.4)	5,296 (4.4)	1,277 (2.0)	2,954 (4.0)	544 (3.3)
Natural greenspace (Km^2)						
Low (tertile 1; <0.15)	217,719 (32.2)	101,143 (31.5)	38,358 (31.8)	22,416 (35.0)	24,280 (33.1)	5,560 (33.8)
Moderate (tertile 2; 0.15 to 0.32)	215,648 (31.9)	104,618 (32.6)	39,775 (32.9)	21,096 (32.9)	24,472 (33.3)	5,344 (32.5)
High (tertile 3; >0.32)	214,343 (31.7)	101,442 (31.6)	37,334 (30.9)	19,271 (30.1)	21,732 (29.6)	4,987 (30.3)
Missing	28,976 (4.3)	14,236 (4.4)	5,296 (4.4)	1,277 (2.0)	2,954 (4.0)	544 (3.3)

Data are presented as mean ± standard deviation or as number (%).

Supplementary Table 4. Descriptive statistics of climatic variables during the study period (2006-22).

	Mean (SD)	Percentiles						
		1st	5th	10th	Median	90th	95th	99th
Mean temperature (°C)								
Warm season	15.9 (2.8)	9.8	11.5	12.4	15.8	19.5	20.7	23.3
Cold season	5.7 (3.3)	-2.2	0.3	1.5	5.8	10	11.1	13.1
TCN (°C)								
Warm season	0.01 (1.8)	-4.6	-3	-2.2	0	2.2	2.9	4.2
Cold season	-0.03 (1.9)	-5.2	-3.4	-2.6	0.1	2.2	2.8	3.9
Rainfall amount (mm)								
Warm season	2.2 (4.7)	0	0	0	0.1	6.8	11.1	22.8
Cold season	2.6 (4.4)	0	0	0	0.6	7.7	11.3	20.3

Supplementary Table 5. Associations of moderately high ambient temperature and moderately positive temperature change between neighbouring days with any-cause and cause-specific hospitalizations.

	Moderately high ambient temperature (90th percentile *vs.* 1st percentile)		Moderately positive temperature change between neighbouring days (TCN) (90th percentile *vs.* 0°C)	
	Warm season	Cold season	Warm season	Cold season
	OR (95% CI)	OR (95% CI)	OR (95% CI)	OR (95% CI)
Any cause	1.01 (0.99, 1.03)	1.04 (1.02, 1.06)	1.01 (1.00, 1.02)	1.02 (1.01, 1.03)
Cardiovascular disease	1.00 (0.98, 1.03)	1.06 (1.03, 1.09)	1.01 (0.99, 1.02)	1.02 (1.01, 1.03)
Respiratory disease	1.04 (0.99, 1.08)	1.05 (1.00, 1.11)	1.00 (0.98, 1.03)	1.02 (0.99, 1.04)
Renal disease	1.07 (1.01, 1.14)	0.98 (0.91, 1.05)	1.02 (0.99, 1.05)	1.02 (0.99, 1.05)
Mental disorders	1.05 (0.99, 1.11)	1.07 (1.00, 1.15)	1.01 (0.98, 1.04)	1.02 (0.99, 1.04)
Heat related illness	1.13 (1.00, 1.27)	1.22 (1.04, 1.43)	1.09 (1.03, 1.16)	1.03 (0.97, 1.09)

Models adjusted for rainfall and public holiday. The odds ratio (OR) and 95% confidence intervals (CI) were calculated for the 90th percentile of ambient temperature distribution in reference to the 1st percentile. For TCN, the ORs and 90% CI were calculated for the 90th percentile in reference to 0°C temperature change from previous day. Warm season refers to periods between June and September, and cold season refers to periods between November and March for each year.

Supplementary Table 6. Associations of high ambient temperature and positive temperature change between neighbouring days over 0-10 lag days with any cause and cause-specific hospitalizations.

	High ambient temperature (95th percentile *vs.* 1st percentile)		Positive temperature change between neighbouring days (TCN) (95th percentile *vs.* 0°C)	
	Warm season	Cold season	Warm season	Cold season
	OR (95% CI)	OR (95% CI)	OR (95% CI)	OR (95% CI)
Any cause	1.00 (0.97, 1.02)	1.08 (1.04, 1.11)	1.03 (1.00, 1.06)	1.05 (1.02, 1.07)
Cardiovascular disease	0.98 (0.94, 1.02)	1.09 (1.03, 1.15)	1.01 (0.97, 1.06)	1.06 (1.02, 1.11)
Respiratory disease	1.00 (0.93, 1.06)	1.06 (0.97, 1.15)	1.03 (0.96, 1.10)	1.07 (1.00, 1.13)
Renal disease	1.13 (1.04, 1.24)	0.91 (0.80, 1.03)	1.07 (0.98, 1.18)	0.99 (0.91, 1.08)
Mental disorders	1.06 (0.97, 1.15)	0.99 (0.87, 1.11)	1.03 (0.95, 1.13)	1.00 (0.92, 1.09)
Heat related illness	1.19 (1.00, 1.42)	0.99 (0.75, 1.30)	1.23 (1.03, 1.46)	1.11 (0.94, 1.32)

Models adjusted for rainfall and public holiday. The odds ratio (OR) and 95% confidence intervals (CI) were calculated for the 95th percentile of ambient temperature distribution in reference to the 1st percentile. For TCN, the OR and 95% CI were calculated for the 95th percentile in reference to 0°C temperature change from previous day. Warm season refers to periods between June and September, and cold season refers to periods between November and February for each year.

Supplementary Table 7. Associations of high ambient temperature and positive temperature change between neighbouring days with any cause and cause-specific hospitalizations in the pre-COVID-19 period (2006-19).

	High ambient temperature (95th percentile *vs.* 1st percentile)		Positive temperature change between neighbouring days (TCN) (95th percentile *vs.* 0°C)	
	Warm season	Cold season	Warm season	Cold season
	OR (95% CI)	OR (95% CI)	OR (95% CI)	OR (95% CI)
Any cause	1.01 (0.99, 1.04)	1.03 (1.01, 1.05)	1.01 (0.99, 1.03)	1.03 (1.02, 1.05)
Cardiovascular disease	0.99 (0.96, 1.03)	1.04 (1.01, 1.08)	1.01 (0.98, 1.03)	1.04 (1.01, 1.06)
Respiratory disease	1.03 (0.97, 1.10)	1.04 (0.98, 1.10)	1.03 (0.99, 1.07)	1.05 (1.01, 1.09)
Renal disease	1.05 (0.97, 1.15)	0.93 (0.86, 1.01)	1.02 (0.96, 1.07)	1.03 (0.98, 1.09)
Mental disorders	1.02 (0.95, 1.10)	1.06 (0.97, 1.15)	0.99 (0.94, 1.04)	1.03 (0.98, 1.08)
Heat related illness	1.09 (0.91, 1.29)	1.30 (1.06, 1.59)	1.11 (0.98, 1.24)	1.12 (1.00, 1.25)

Models adjusted for rainfall and public holiday. The odds ratio (OR) and 95% confidence intervals (CI) were calculated for the 95th percentile of ambient temperature distribution in reference to the 1st percentile. For TCN, the OR and 95% CI were calculated for the 95th percentile of TCN distribution in reference to 0°C. Warm season refers to periods between June and September, and cold season refers to periods between November and February for each year.

Supplementary Table 8. Associations of high ambient temperature and positive temperature change between neighbouring days with any cause and cause-specific hospitalizations during the COVID period (2020-2022).

	High ambient temperature (95th percentile *vs.* 1st percentile)		Positive temperature change between neighbouring days (TCN) (95th percentile *vs.* 0°C)	
	Warm season	Cold season	Warm season	Cold season
	OR (95% CI)	OR (95% CI)	OR (95% CI)	OR (95% CI)
Any cause	1.02 (0.98, 1.05)	1.11 (1.03, 1.19)	1.01 (0.99, 1.04)	1.01 (0.98, 1.05)
Cardiovascular disease	1.02 (0.98, 1.07)	1.16 (1.06, 1.28)	1.01 (0.98, 1.05)	1.02 (0.98, 1.07)
Respiratory disease	1.06 (0.99, 1.15)	1.14 (0.98, 1.33)	0.97 (0.92, 1.03)	0.98 (0.92, 1.05)
Renal disease	1.13 (1.03, 1.25)	1.09 (0.90, 1.32)	1.04 (0.96, 1.11)	1.02 (0.94, 1.11)
Mental disorders	1.07 (0.97, 1.18)	1.17 (0.98, 1.41)	1.08 (1.00, 1.16)	1.05 (0.97, 1.13)
Heat related illness	1.15 (1.13, 1.61)	1.07 (0.78, 1.46)	1.15 (1.01, 1.30)	0.96 (0.83, 1.10)

Models adjusted for rainfall and public holiday. The odds ratio (OR) and 95% confidence intervals (CI) were calculated for the 95th percentile of ambient temperature distribution in reference to the 1st percentile. For TCN, the OR and 95% CI were calculated for the 95th percentile of TCN distribution in reference to 0°C. Warm season refers to periods between June and September, and cold season refers to periods between November and February for each year.

Supplementary Table 9. Associations of high ambient temperature and positive temperature change between neighbouring days over lag days 0-3 with any cause and cause-specific first hospitalization record for each individual participant.

	High ambient temperature (95th percentile *vs.* 1st percentile)		Positive temperature change between neighbouring days (TCN) (95th percentile *vs.* 0°C)	
	Warm season	Cold season	Warm season	Cold season
	OR (95% CI)	OR (95% CI)	OR (95% CI)	OR (95% CI)
Any cause	1.02 (0.97, 1.07)	1.05 (1.00, 1.10)	1.04 (1.00, 1.07)	1.03 (1.00, 1.06)
Cardiovascular disease	1.00 (0.97, 1.03)	1.09 (1.04, 1.14)	1.01 (0.99, 1.03)	1.03 (1.01, 1.05)
Respiratory disease	1.04 (0.99, 1.08)	1.08 (1.00, 1.16)	1.01 (0.98, 1.04)	1.03 (1.00, 1.06)
Renal disease	1.09 (1.02, 1.16)	0.96 (0.87, 1.06)	1.03 (0.98, 1.07)	1.03 (0.98, 1.08)
Mental disorders	1.04 (0.98, 1.11)	1.12 (1.01, 1.24)	1.02 (0.97, 1.06)	1.04 (0.99, 1.08)
Heat related illness	1.18 (1.05, 1.34)	1.33 (1.06, 1.67)	1.12 (1.03, 1.22)	1.05 (0.96, 1.15)

Models adjusted for rainfall and public holiday. The odds ratio (OR) and 95% confidence intervals (CI) were calculated for the 95th percentile of ambient temperature distribution in reference to the 1st percentile. For TCN, the OR and 95% CI were calculated for the 95th percentile of TCN distribution in reference to 0°C. Warm season refers to periods between June and September, and cold season refers to periods between November and February for each year.

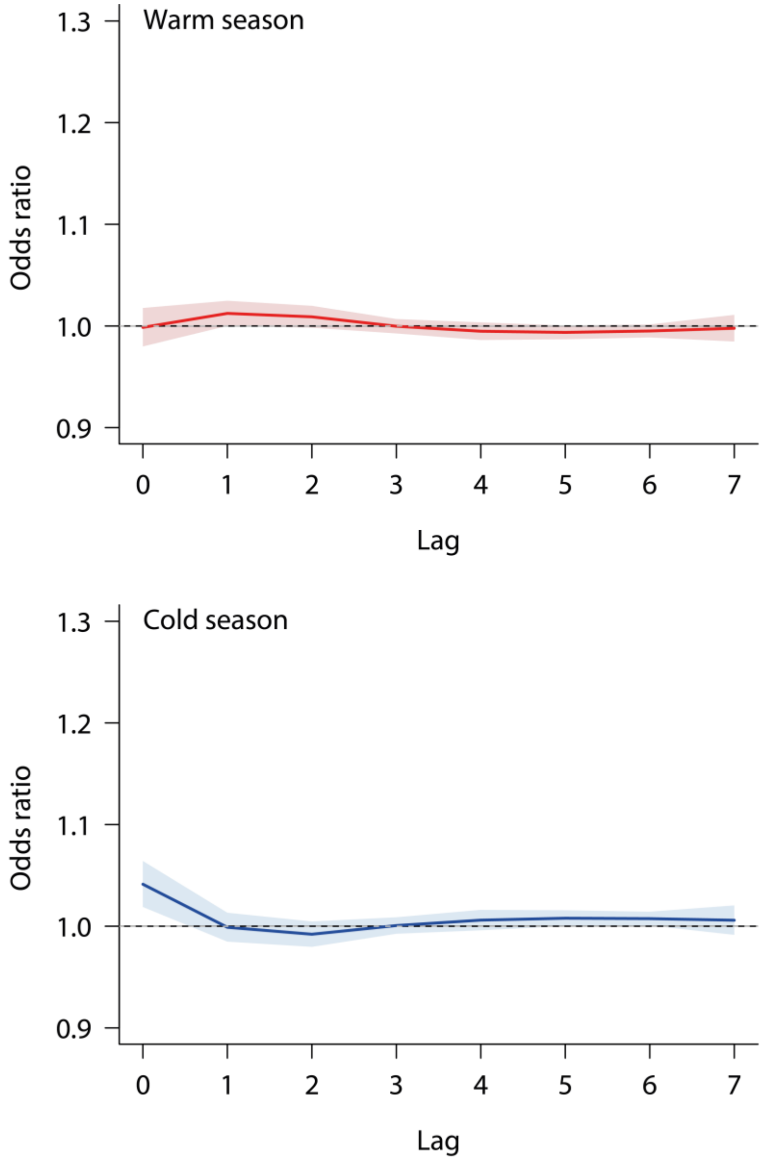

Supplementary Figure 1. Overall lag structure in effects of high ambient temperature on hospitalizations for any cause measured over 0-7 lag days during warm season (red), and cold season (blue). Models adjusted for rainfall and public holiday. Solid lines represent mean odds ratios of hospitalizations, whereas shaded areas represent 95% CI.

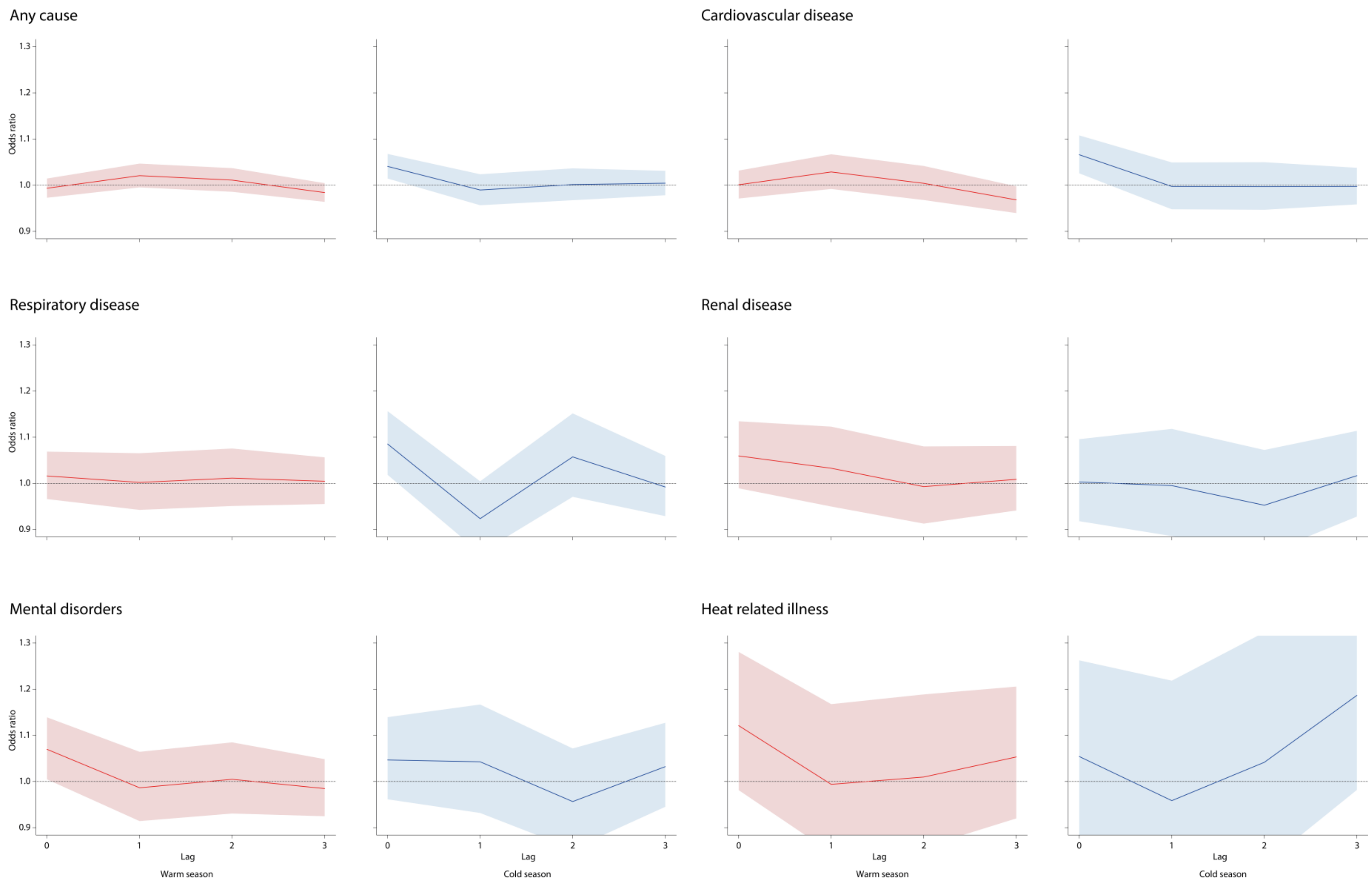

Supplementary Figure 2. Overall lag structure in effects of high ambient temperature on hospitalizations for any cause, cardiovascular disease, respiratory disease, renal disease, mental disorders and heat related illness measured over 0-3 lag days during warm season (red), and cold season (blue). Models adjusted for rainfall and public holiday. Solid lines represent mean odds ratios of hospitalizations, whereas shaded areas represent 95% CI.

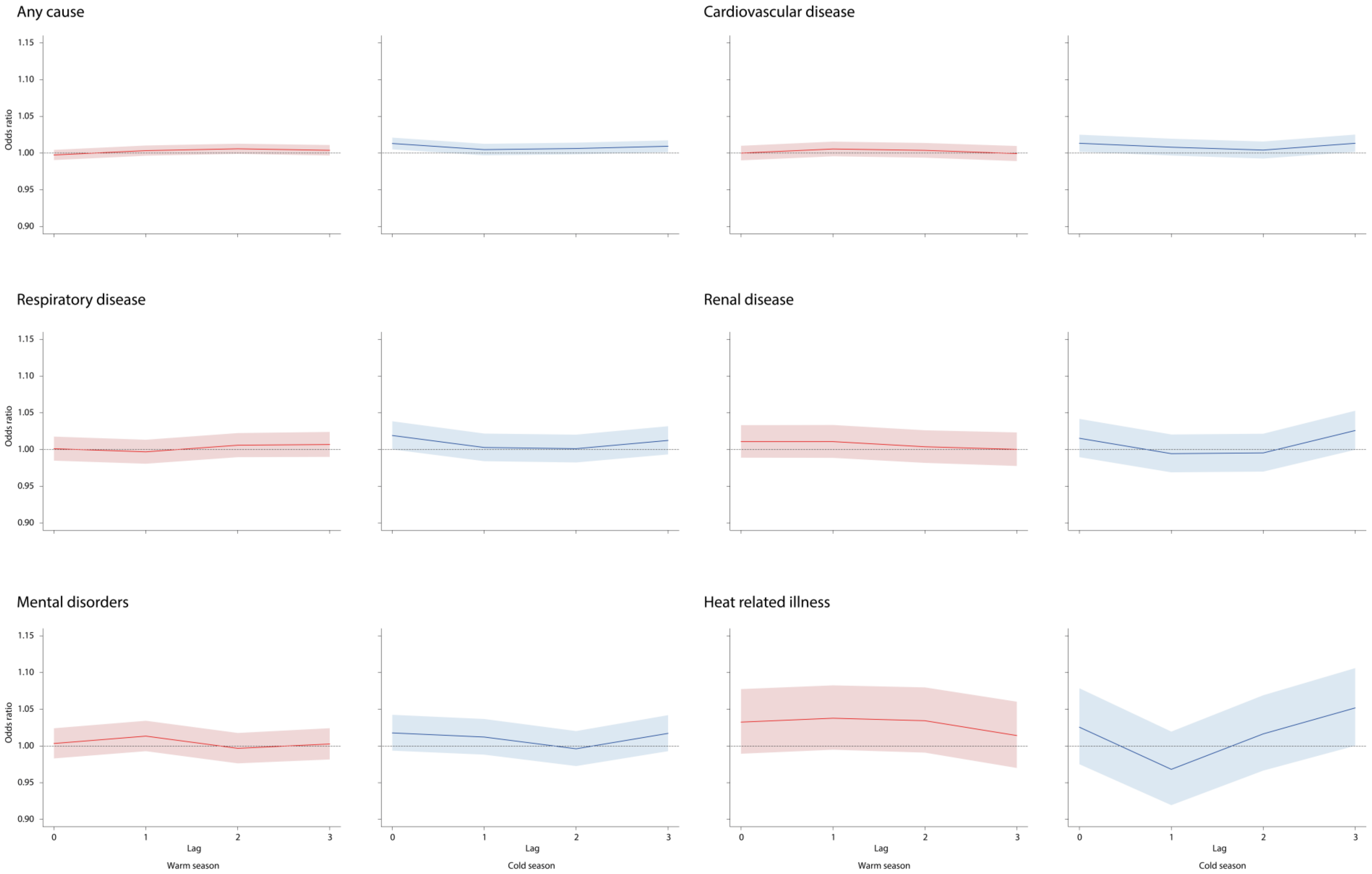

Supplementary Figure 3. Overall lag structure in effects of positive temperature change in neighbouring days (TCN) on hospitalizations for any cause, cardiovascular disease, respiratory disease, renal disease, mental disorders and heat related illness measured over 0-3 lag days across during warm season (red), and cold season (blue). Models adjusted for rainfall and public holiday. Solid lines represent mean odds ratios of hospitalizations, whereas shaded areas represent 95% CI.

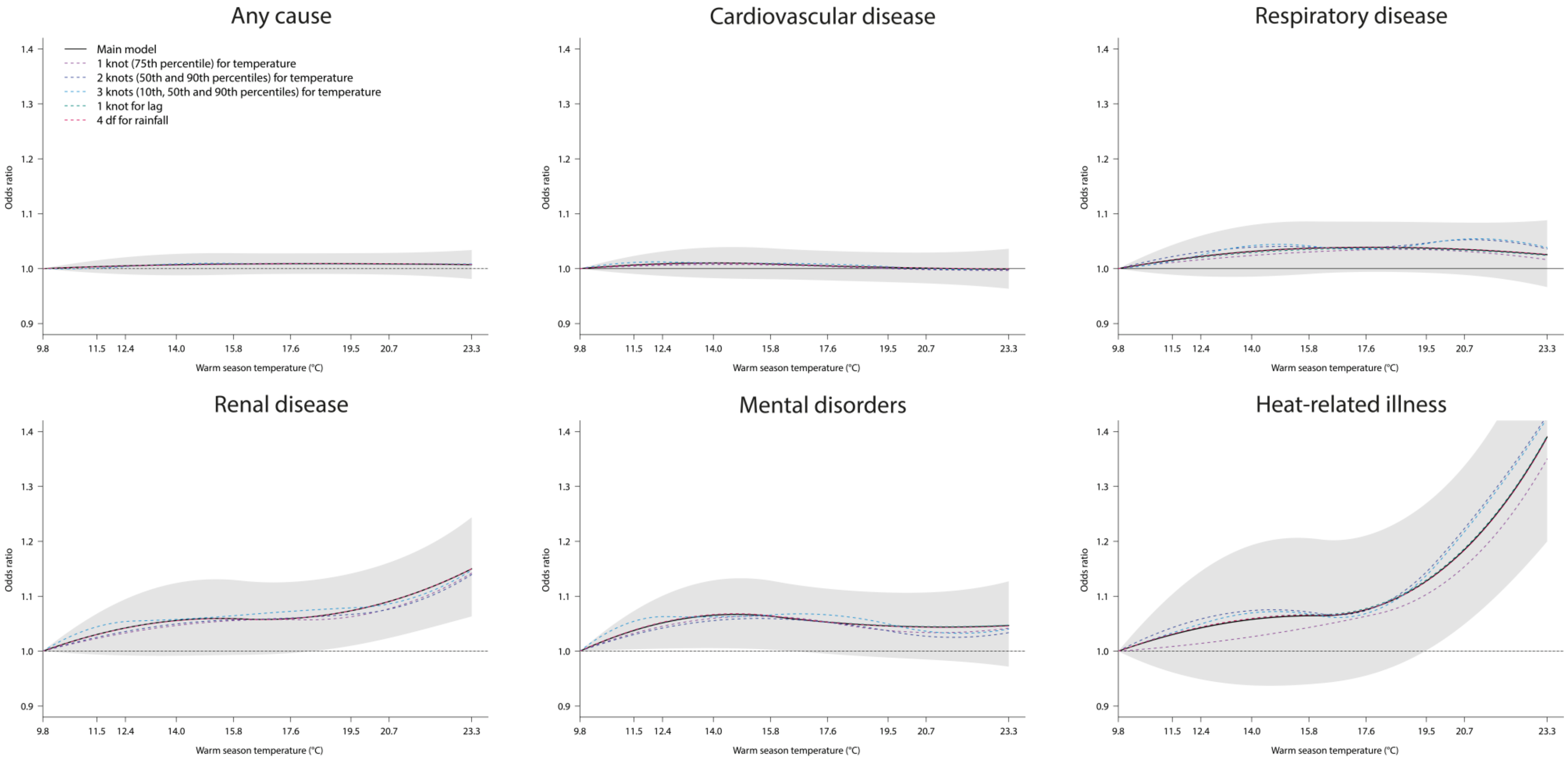

Supplementary Figure 4. Sensitivity analysis using alternative modeling parameters for mean temperature during warm season. Cumulative exposure-response curves for the associations of daily mean ambient temperature with hospitalizations for any cause, cardiovascular disease, respiratory disease, renal disease, mental disorders and heat related illness during warm season. Model adjusted for rainfall and public holiday. The odds ratio (OR) and 95% confidence intervals (CI) were calculated in reference to the 1st percentile of ambient temperature distribution. Shaded areas represent 95% CI.

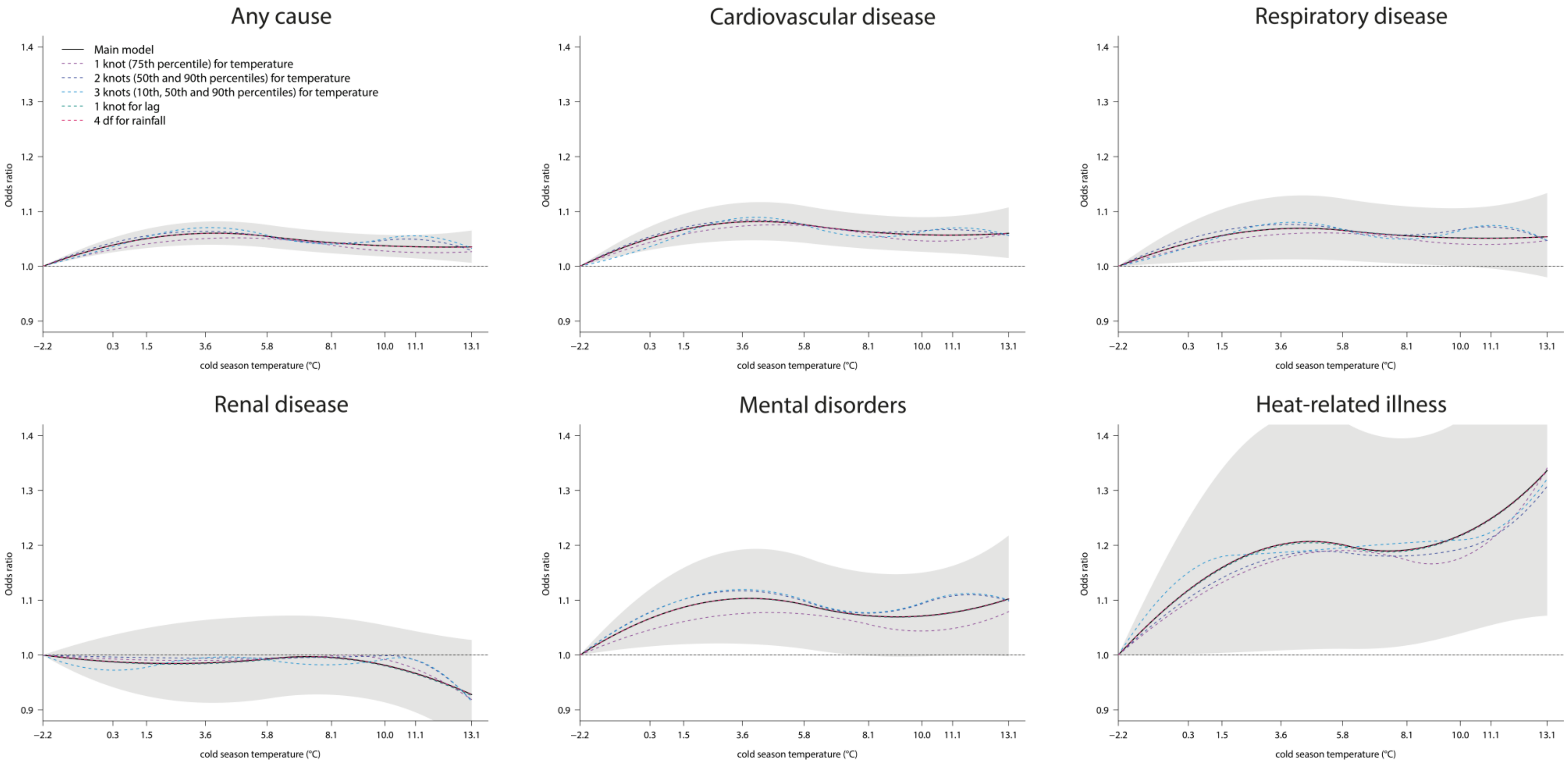

Supplementary Figure 5. Sensitivity analysis using alternative modeling parameters for mean temperature during cold season. Cumulative exposure-response curves for the associations of daily mean ambient temperature with hospitalizations for any cause, cardiovascular disease, respiratory disease, renal disease, mental disorders and heat related illness during cold season. Model adjusted for rainfall and public holiday. The odds ratio (OR) and 95% confidence intervals (CI) were calculated in reference to the 1st percentile of ambient temperature distribution. Shaded areas represent 95% CI.

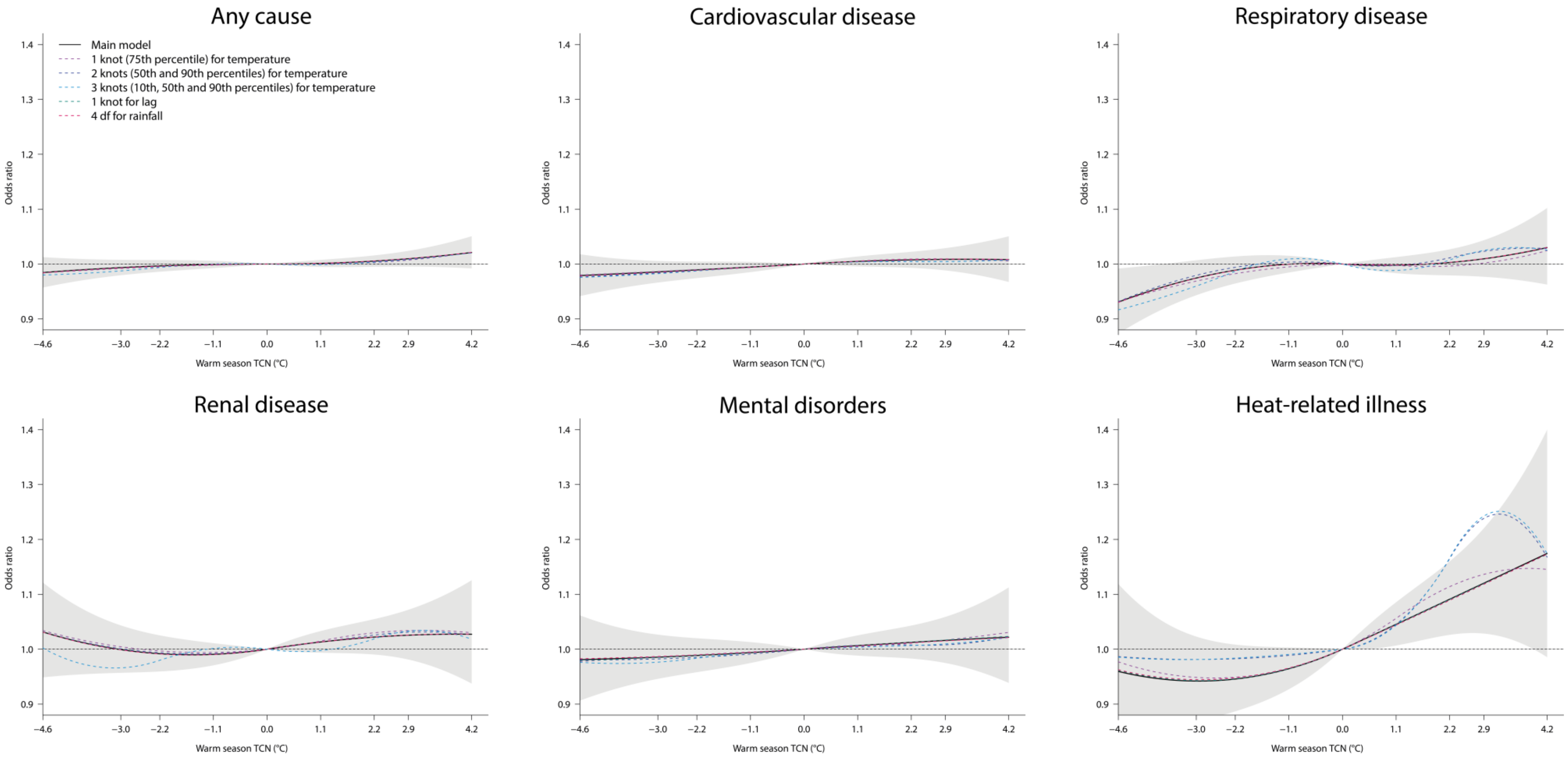

Supplementary Figure 6. Sensitivity analysis using alternative modeling parameters for temperature change between neighbouring days (TCN) during warm season. Cumulative exposure-response curves for the associations of warm season TCN with hospitalizations for any cause, cardiovascular disease, respiratory disease, renal disease, mental disorders and heat related illness during warm season. Models adjusted for rainfall and public holiday. The OR and 95% CI were calculated in reference to 0°C. Shaded areas represent 95% CI.

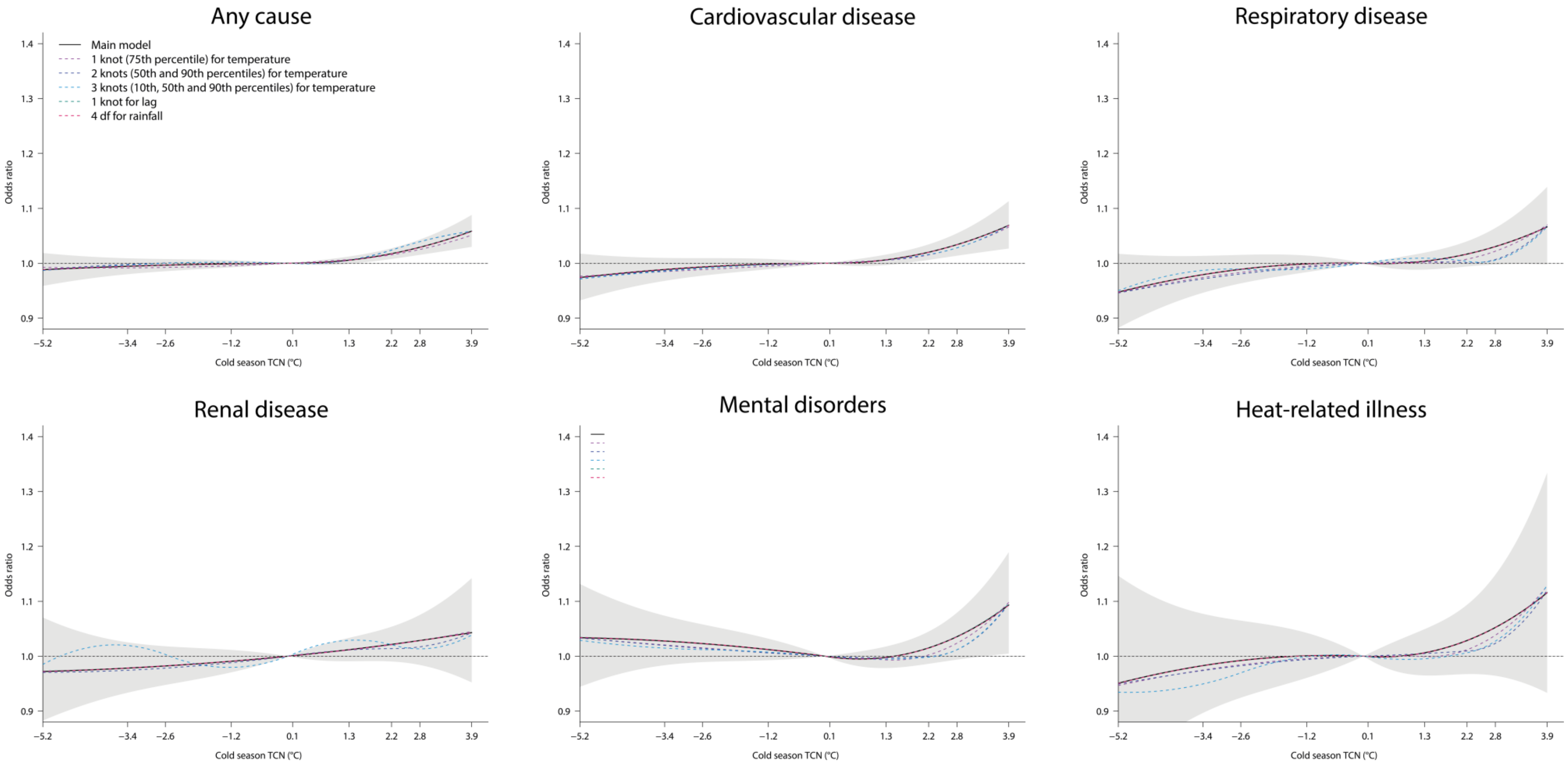

Supplementary Figure 7. Sensitivity analysis using alternative modeling parameters for temperature change between neighbouring days (TCN) during cold season. Cumulative exposure-response curves for the associations of cold season TCN with hospitalizations for any cause, cardiovascular disease, respiratory disease, renal disease, mental disorders and heat related illness during cold season. Model adjusted for rainfall and public holiday. The OR and 95% CI were calculated in reference to 0°C. Shaded areas represent 95% CI.

References

1 Fried, L. P. *et al.* Frailty in Older Adults: Evidence for a Phenotype. *The Journals of Gerontology: Series A* **56**, M146-M157, doi:10.1093/gerona/56.3.M146 (2001).

2 Hanlon, P. *et al.* Frailty and pre-frailty in middle-aged and older adults and its association with multimorbidity and mortality: a prospective analysis of 493 737 UK Biobank participants. *The Lancet Public Health* **3**, e323-e332, doi:10.1016/S2468-2667(18)30091-4 (2018).

3 Sarkar, C., Webster, C. & Gallacher, J. UK Biobank Urban Morphometric Platform (UKBUMP)–a nationwide resource for evidence-based healthy city planning and public health interventions. *Annals of GIS* **21**, 135-148 (2015).

4 Lai, K. Y., Kumari, S., Gallacher, J., Webster, C. & Sarkar, C. Nexus between residential air pollution and physiological stress is moderated by greenness. *Nature Cities* **1**, 225-237, doi:10.1038/s44284-024-00036-6 (2024).
