# Supplementary material for: Ambient temperature and the variability between neighbouring days impacts in-patient hospitalizations in the United Kingdom
Source: Commun Med (Lond). 2026 Jan 26;6:90. doi: 10.1038/s43856-025-01355-y (PMC12881400; doi:10.1038/s43856-025-01355-y)
Supplement: Supplementary file 2 — Description of Additional Supplementary Data [file 43856_2025_1355_MOESM2_ESM.docx]

Description of additional supplementary file

File name: Supplementary Data 1

Description: Associations of high ambient temperature and positive temperature change between neighbouring days with any cause and cause-specific hospitalizations based on temperature percentiles derived from specific UK climatic regions.

File name: Supplementary Data 2

Description: Subgroup analysis for associations of high temperature and positive temperature change between neighbouring days (TCN) with overall hospitalization (any cause) during warm season.

File name: Supplementary Data 3

Description: Subgroup analysis for associations of high temperature and positive temperature change between neighbouring days (TCN) with overall hospitalization (any cause) during cold season.

File name: Supplementary Data 4

Description: Literature review of 22 case-crossover studies examining the associations between ambient temperature and hospitalizations published in recent 5 years.
